# Modeling studies of the adsorption of Methyl Red and Acid Yellow 36 dyes by sulphonated *Ulva lactuca* carbon

**DOI:** 10.1038/s41598-025-29983-3

**Published:** 2025-12-10

**Authors:** Mohamed A. Hassaan, Murat Yılmaz, Amany El Sikaily, Amany G. M. Shoaib, Mohamed A. El-Nemr, Ahmed El Nemr

**Affiliations:** 1 Environment Division, National Institute of Oceanography and Fisheries (NIOF), Kayet Bey, Elanfoushy, Alexandria Egypt; 2 Bahçe Vocational School, Department of Chemistry and Chemical Processing Technologies, Osmaniye Korkut Ata University, Osmaniye, 80000 Türkiye Turkey; 3 Department of Chemical Engineering, Faculty of Engineering, Minia University, Minia, 61519 Egypt; 4 The Higher Canal Institute of Engineering and Technology, Al Salam 1 - Abu Bakr Al Siddiq Street, Suez, Egypt

**Keywords:** Adsorption kinetics, Isotherm modelling, Azo dye removal, Biomass-derived sulphonated carbon, *Ulva lactuca* biochar, Chemistry, Environmental sciences, Materials science

## Abstract

This study investigated the adsorption of Acid Yellow 36 (AY36) and Methyl Red (MR) dyes using a novel sulfonated biochar carbon (GASC) synthesized from green algae (*Ulva lactuca*) via a reflux method in the presence of 80% sulfuric acid (H_2_SO_4_). The structural and surface characteristics of the resultant GASC were evaluated by Fourier transform infrared spectroscopy (FTIR), Brunauer–Emmett–Teller (BET) surface area analysis, scanning electron microscopy (SEM), and thermogravimetric analysis (TGA). GASC exhibited a relatively low BET surface area of 6.27 m²/g, with a monolayer volume of 1.4392 cm³ (STP)/g. The total pore volume was measured as 0.0203 cm³/g, and the mean pore size was 12.971 nm. GASC’s total weight loss was 46.52%, indicating higher thermal stability compared to GAUL (78.01%). FTIR spectra validated the existence of many functional groups on GASC. Adsorption experiments for AY36 and MR dyes were conducted under varying conditions, including initial dye concentrations (50–150 mg/L), contact times (5–180 min), solution pH (2–12), and GASC dosages (0.5–1.5 g/L). According to the Langmuir isotherm, the maximum adsorption capacities (*Q*_*m*_​) of GASC for AY36 and MR dyes were 216.45 and 454.55 mg g^–1^, respectively. Kinetic assessments indicated that the adsorption of both dyes was optimally represented by the pseudo-second order model, validating the efficacy of the synthesized GASC as an adsorbent for eliminating AY36 and MR dyes from aqueous solutions. Furthermore, response surface methodology (RSM) and Artificial neural networks (ANN) were applied to model and optimize adsorption. Optimization of the adsorption parameters using the RSM study concluded that a maximum AY-36 dye removal percentage of 96.76% could be reached when employing 147.41 mg of GASC and 56.08 mg/L of AY-36 dye solution. The most significant degradation percentage of MR dye, 99.94%, was achieved using 132.34 mg of GASC and 59.30 mg/L of MR dye solution.

**Supplementary Information:**

The online version contains supplementary material available at 10.1038/s41598-025-29983-3.

## Introduction

In the 21 st century, water pollution has emerged as a critical global concern, primarily driven by rapid industrialization, urban expansion, and the unsustainable exploitation of natural resources, which pose significant threats to both environmental ecosystems and human health^1^. Synthetic dyes are recognized as major environmental pollutants due to their extensive application across various sectors, such as textiles, printing, paper production, food manufacturing, cosmetics, and leather processing^2,3^. Synthetic dyes are essential to the textile industry, contributing significantly to the coloration and aesthetic appeal of fabrics. Nevertheless, their widespread use is accompanied by serious environmental and health concerns. With more than 10,000 different types of synthetic dyes produced globally and an estimated annual output approaching 800,000 tons, a considerable amount is discharged into the environment through wastewater, posing substantial ecological risks^4,5^. These dyes are typically categorized into three groups based on their dissociation properties in aqueous media: anionic dyes (including acid, direct, and reactive types), cationic dyes (primarily basic dyes), and nonionic dyes (such as disperse dyes)^6^.

Metanil Yellow, also referred to as Acid Yellow 36 dye, is a commonly used azo dye primarily applied in the textile industry for dyeing protein-based fibers such as wool and silk. In addition to its use in textiles, AY36 dye has been utilized in products like soaps, shoe polishes, laundry and cleaning agents, and pigments. Despite its application in certain food items, including sweets, juices, and ice cream, its use in the food industry is strictly banned due to its recognized carcinogenic properties^7,8^. Methyl Red (MR) dye, an anionic azo dye, is widely utilized in both industrial applications and laboratory analyses. However, its release into aquatic environments poses significant health risks, including gastrointestinal tract irritation, eyes, and skin^9–11^. The presence of benzene rings in its structure contributes to its mutagenic potential under aerobic conditions and limits its biodegradability^12^. Due to these concerns, effective removal of MR dye from contaminated water prior to environmental discharge is essential.

Various techniques have been developed for dye removal, such as photocatalytic degradation^13^, membrane filtration^14,15^, Fenton-like reaction^16^, biological treatments^17,18^, advanced oxidation processes^19^, and adsorption^20,21^. Among these, adsorption is particularly notable owing to its simplicity, cost-effectiveness, high efficiency, broad applicability, and minimal generation of secondary pollutants. This method has been extensively utilized to remediate dye-contaminated real wastewater samples^22–24^.

Activated carbon is extensively recognized as a primary adsorbent in numerous dye elimination processes^25,26^. Nevertheless, its practical application is often limited by high production costs and difficulties in regeneration^27,28^. To address these limitations, low-cost precursors such as spinach waste^29^, spent coffee grounds^30^, watermelon peel^31^, palm petiole^32^, date palm kernel^33^, cocoa pods^34^, mandarin peel^20^, sawdust^35^, rice husk^36^, red algae^37^, and pea pods^38^ have been investigated as alternative sources for carbon-based adsorbents. These materials have demonstrated potential for economically eliminating various contaminants from wastewater.

In addition, recent studies on machine learning approaches to dye removal have appeared in the literature. Kumari at all. (2025) reported the effectiveness of artificial neural networks (ANNs), response surface methodology (RSMs), adaptive neuro-fuzzy inference systems (ANFISs), and k-nearest neighbors (kNNs) in removing crystal violet (CV) from wastewater. Statistical analysis of errors was performed, and they reported a maximum removal efficiency of 97.46% under optimized settings. They also found that while all models were accurate in predicting CV dye removal, the ANN model exhibited higher accuracy compared to the other models^39^. In another study, Kumari et al. (2023) compared the predictive capabilities of RSM and ANN for adsorption treatment of MB and CV dye simulated wastewater using Saccharum officinarum L and reported using a specific mechanism that led to significant maximum removals of 98.3% and 98.2% for dyes (MB and CV), respectively^40^. In another study using RSM, Rasouli and co-workers synthesized a highly efficient α-Fe_2_O_3_@TiO_2_ nanocomposite for the removal of cefixime using a rapid sonochemical technique and a conventional wet impregnation method. The effects of operational parameters on obtaining the optimum photocatalyst were investigated. They reported a maximum cefixime degradation of 98.8% under optimum conditions using RSM^41^.

The adsorption efficiency of carbon-based materials in wastewater treatment is strongly influenced by the type and quantity of surface functional groups. This can be tailored through chemical surface modification. Nanoscale engineering, oxidation, surface activation, and metal incorporation have also improved adsorption performance^42^. Enhancing the surface chemistry via treatment with various acids (e.g., H_3_PO_4_, H_2_SO_4_, HNO_3_), bases (e.g., NaClO, KOH), or oxidizing agents (e.g., NH_3_.H_2_O, (NH_4_)_2_S_2_O_8_, H_2_O_2_, KmnO_4_, NaClO) can increase the density of functional groups on the carbon surface^43–45^.

In this study, green algae (*Ulva lactuca*) (GAUL) were selected as a precursor for synthesizing highly porous carbon materials. Employing sulfonated biochar carbon produced from green algae *Ulva lactuca* for pollutant removal from aqueous media offers an innovative and ecologically sustainable approach to environmental remediation. As a carbon material precursor, green algae constitute a renewable and eco-friendly resource, capable of being cultivated in diverse aquatic environments, including those unfit for conventional applications^46–49^. Modified algae-derived biochar is characterized by its enhanced surface area and porosity, which contribute significantly to its effectiveness in adsorbing diverse contaminants, including heavy metals, dyes, and organic substances, from wastewater sources^50–52^. Additionally, incorporating nitrogen-containing functional groups further improves the surface chemistry and adsorption performance of algae-based biochar^53^. These results highlight the promising potential of chemically modified biochar derived from green algae for efficient contaminant removal.

No studies have investigated the adsorption performance of sulphonated carbon materials synthesized via H₂SO₄ activation under a reflux system using green algae (*Ulva lactuca*) as a precursor for the removal of AY36 and MR dyes from wastewater. Although AY36 and MR dye are classified as anionic dyes, they exhibit distinct molecular structures, functional groups, and physicochemical properties. By investigating the adsorption behavior of both dyes, this study aims to provide a comprehensive assessment of the GASC efficacy and selectivity toward a diverse range of anionic dye contaminants. This dual-dye approach facilitates a deeper understanding of how structural and chemical differences influence adsorption mechanisms, thereby enhancing the evaluation of GASC as a versatile and effective adsorbent for treating industrial wastewater containing various anionic dyes.

This study aims to evaluate the adsorption efficiency of GASC, a low-cost adsorbent derived from dried *Ulva lactuca*, to eliminate AY36 and MR dyes from aqueous solutions. Key parameters examined in the adsorption process include initial dye concentration, solution pH, contact time, and GASC dosage. Kinetic and isotherm models were employed to elucidate the fundamental adsorption mechanism and to assess the maximum adsorption capacities. Furthermore, RSM was utilized to optimize the adsorption conditions for both dyes on the GASC adsorbent.

## Materials and methods

### Materials

#### Chemicals

Sulfuric acid (H_2_SO_4_ (99%), Mwt = 98.079 g/mole), Sodium Hydroxide (NaOH, Mwt = 39.997 g/mole), Sodium bicarbonate NaHCO_3_ (Mwt = 84 g/mole), Hydrogen Chloride (HCl, Mwt = 36.46 g/mole), Acid Yellow 36 dye (Synonyms: Metanil Yellow, MF = C_18_H_14_N_3_NaO_3_S, Mwt = 375.38 g/mole, CAS No.: 587-98-4) were purchased from Sigma-Aldrich, Germany. Methyl Red dye (Synonyms: O-Methyl Red, Acid Red 2, MF = C_15_H_15_N_3_O_2_, Mwt = 269.30 g/mole, CAS No.: 587-98-4) was purchased from Sigma-Aldrich, Germany.

#### Instrument

The experimental setup included a JSOS-500 shaker, a digital UV–Visible spectrophotometer, and a JENCO-6173 pH meter.

#### Sampling

Green algae (*Ulva lactuca*) (GAUL) were collected from the coastal zone of Alexandria, meticulously rinsed with tap water, followed by distilled water, and air-dried overnight at ambient temperature. The samples were then dried in an oven at 105 °C and pulverized into a fine powder using an electric blender. The resulting powder was subjected to sieving via a 100-mesh screen prior to utilization. The bulk density, ash content, and moisture content of the prepared *U. lactuca* biomass were determined to be 0.8179 g/mL, 35.12%, and 15.3%, respectively.

#### Preparation of stock solution

**Fig. 1:**
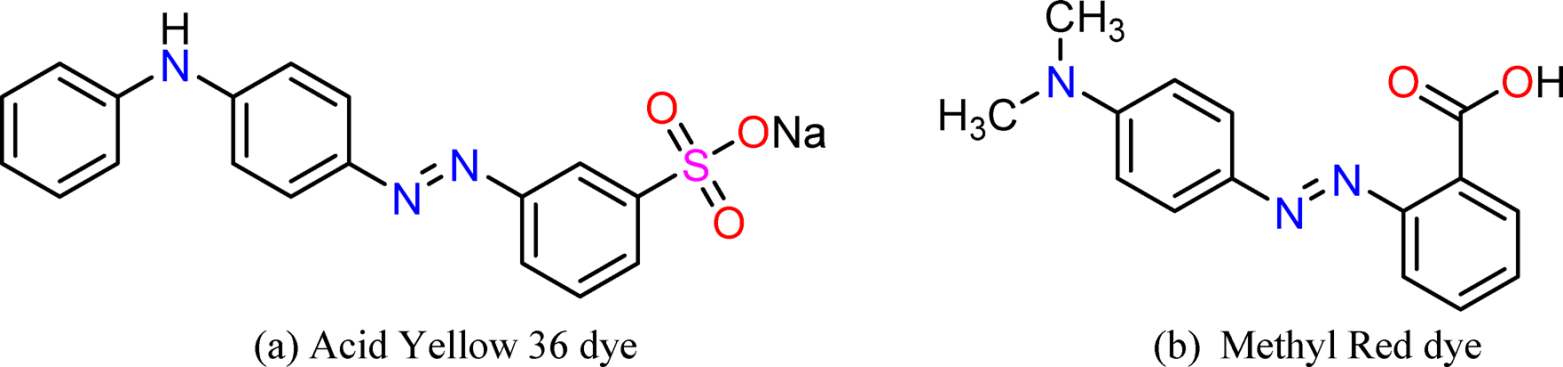
The chemical structure of .

 Stock solutions of AY36 and Methyl Red (MR) dyes were prepared by dissolving 1.0 g of each dye in 1000 mL of distilled water (DW), yielding 1000 mg/L concentration. These stock solutions were diluted to the desired concentrations for standard calibration curves and adsorption experiments. A double-beam UV–Visible spectrophotometer (PG Instruments, model T80, United Kingdom), equipped with 1 cm path length glass cells, was utilized for all absorbance measurements. Figure 1 depicts the chemical structure of both dyes.

### Preparation of GASC

GAUL was rinsed with distilled water (DW), subjected to oven drying at 120 °C for 72 h, and then pulverized into a fine powder. A total of 300 g of the dried GAUL powder was introduced to 600 mL of concentrated H_2_SO_4_, subsequently followed by the incorporation of 250 mL of distilled water in a reflux system at 350 °C for 4 h. The resulting mixture was filtered and rinsed with DW. To remove any residual acid, the solid was treated with a 1% NaHCO₃ solution until the filtrate attained a neutral pH. The product was subsequently rinsed with ethanol and dried at 120 °C for 24 h. The resulting material is called sulfonated biochar carbon (GASC)^54^.

### Batch absorption procedure

The removal efficiency of AY36 and Methyl Red (MR) dyes by sulfonated biochar carbon was evaluated through a series of batch adsorption experiments. The influence of starting dye concentration, adsorbent dosage, contact time, and pH on adsorption capacity was examined under batch equilibrium circumstances. Experiments were conducted in 100 mL Erlenmeyer flasks containing varying concentrations of AY36 and MR dye solutions (50, 75, 100, 125, and 150 mg/L) and different dosages of GASC (0.5, 0.75, 1.0, 1.25, and 1.5 g/L) at 25 °C. The flasks were stirred in an isothermal shaker at a constant speed of 200 rpm until equilibrium was attained. Dye concentrations were determined using a double-beam UV–Visible spectrophotometer (PG Instruments, model T80, United Kingdom). The maximum absorbance wavelengths (*λ*_max_) for AY36 and MR dyes were 434 and 492 nm, respectively.

To investigate the impact of initial adsorbate concentration and contact duration on adsorption performance, dye solutions with concentrations ranging from 50 to 150 mg/L for AY36 and Methyl Red (MR) dye were prepared. The influence of solution pH on the adsorption process was assessed by adjusting the initial pH of the solutions from 2 to 12. Adsorption isotherm studies were conducted by fitting the equilibrium data to the Langmuir (LIM), Freundlich (FIM), Temkin (TIM), Dubinin–Radushkevich (DRIM), and Generalized ısotherm (GIM) models. Kinetic studies were performed using pseudo-first-order (PFOM), pseudo-second-order (PSOM), Elovich (EM), Intraparticle diffusion (IPDM), and Film diffusion (FDM) models to describe the adsorption kinetics. For each model, the least-squares correlation coefficient (*R*²), slope, intercept, and both computed (*q*_cal_) and experimental (*q*_exp_) adsorption capacities were determined to evaluate the best fit. The equilibrium adsorption capacities (*q*_*e*_) were predicted by Eq. (1)^55^.

1 $$\:\:{q}_{e}=\frac{\left({C}_{0}-{C}_{e}\right)\:V}{m}$$

where the adsorption capacity (*q*_e_, mg absorbate/g absorbent) represents the ability of the adsorbent to eliminate AY36 and MR dyes from solution at equilibrium. Here, *C*_0_ (mg/L) represents the starting concentration of the dye solution, while *C*_e_ (mg/L) denotes to the dye concentration remaining in solution after a specified contact time. Equation (2) was employed to calculate the percentage removal of AY36 and MR dyes from aqueous solutions.

2 $$\:Removal\:\%\:=\frac{\left({C}_{0}-{C}_{e}\right)\:}{{C}_{0}}\:\times\:100$$

### Characterization

The physicochemical properties of GASC were characterized using the Brunauer–Emmett–Teller (BET) surface area analysis, scanning electron microscopy (SEM), X-ray diffraction (XRD), energy-dispersive X-ray spectroscopy (EDX), proximate analysis, and Fourier transform infrared (FTIR) spectroscopy. The XRD pattern of the dried GASC powder was obtained utilizing an X-ray diffractometer (Ultima IV, Rigaku, Tokyo, Japan) over a *2θ* range. The surface morphology and particle size of the sulfonated biochar carbon were examined using a field emission scanning electron microscope (SEM: LEO, 1450 VP) operated at an accelerating voltage of 15.0 kV. Elemental composition, including carbon (C), nitrogen (N), sulphur (S) and oxygen (O), was analyzed using EDX analysis. The specific surface area, pore size distribution, and pore volume of GASC were determined via nitrogen adsorption–desorption isotherms using the Brunauer–Emmett–Teller (BET) method (BELSORP-Mini II, BEL Japan, Inc.). The surface functional groups of the samples were identified using a Fourier-transform infrared (FTIR) spectrometer (VERTEX 70, equipped with an ATR unit, model V-100) in the spectral range of 400–4000 cm^–1^. Proximate analysis of GASC was performed using a thermogravimetric analyzer (TGA, model SDT650). The TGA data determined the percentages of moisture, fixed carbon, volatile matter, and ash, with the total normalized to 100%. GASC samples were heated in a nitrogen atmosphere from room temperature to 110 °C to assess the moisture content to guarantee thorough dehydration. Subsequently, thermal decomposition was conducted from 25 °C to 1000 °C at a constant heating rate of 5 °C/min under a nitrogen atmosphere. The Thermo Fisher Scientific K-Alpha XPS was used for the X-ray Photoelectron Spectroscopy (XPS) investigation. The study employed a base pressure of around 10^–9^ millibars (mbar) and a pass energy of 50 electron volts (eV). The CHNS/O elemental analysis was conducted using the Thermo Scientific FlashSmart Elemental Analyzer (EA), linked to the Thermo Scientific MAS Plus Autosampler and EagerSmart Data Handling Software^27,38^.

### Response surface methodology (RSM)

Response surface methodology (RSM) was applied to optimize the parameters influencing the adsorption of AY-36 and MR dyes onto GASC. For this purpose, the Box–Behnken design (BBD) was implemented using Design-Expert software (version 13.0.5.0). Initial dye concentrations, adsorbent dosage, solution pH, and contact time were selected as the independent variables^56–59^. The experimental factors and their corresponding levels are summarized in Table 1. The study focused on the removal efficiency (%) of AY36 and MR dyes as the response variables. Thirty experimental runs were done utilizing various combinations of the chosen parameters^60–65^. The detailed experimental design is outlined in Table 1.

**Table 1:** Parameter ranges examined in the optimization process.

Factor	Name	Units	Minimum	Maximum	Mean	Std. Dev.
A	Dosage	g/L	0.5	1.50	1.0	0.3536
C	AY-36 and MR dyes Conc.	mg/L	50	150	150	35.36
B	Time	min	30	150	90	42.43

### Artificial neural networks (ANN) modelling

The feed-forward back-propagation neural network (BPNN) is the most widely used form of artificial neural networks (ANNs). A typical BPNN architecture consists of an input layer (IL) representing the independent variables, one or more hidden layers (HLs), and an output layer (OL) corresponding to the dependent variable. The hidden layers play a crucial role in capturing and modelling the complex nonlinear interactions between inputs and outputs. ANN modelling, drawing inspiration from the architecture of the human brain, forecasts correlations between input and output data via interconnected neurons that process and retain information. This study built a BPNN framework in MATLAB R2015b utilizing the Levenberg–Marquardt (LM) training method, with the dataset partitioned into 70% for training, 15% for validation, and 15% for testing. The optimal ANN architecture consisted of a hidden layer with nine neurons for AY36 dye and six for MR dye, yielding the highest coefficient of determination (*R*²) and the lowest mean square error (MSE). The input parameters considered were the GASC adsorbent dosage (g/L), contact time (min), and initial concentrations of AY36 and MR dyes (mg/L). The dependent variable was the removal of AY36^66–70^.

## Results and discussion

### Physico-chemical studies of GASC

**Fig. 2:**
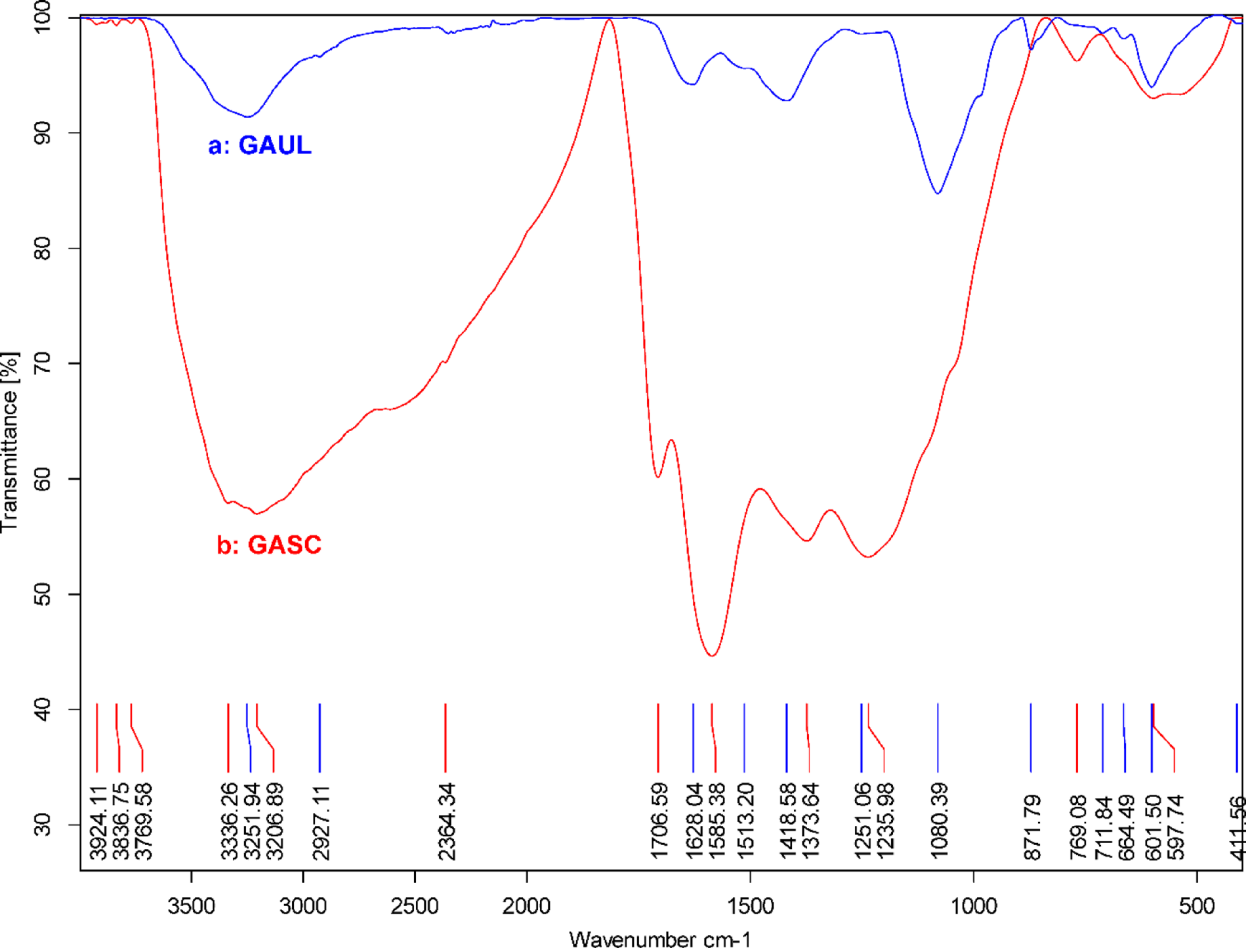
Comparison of FTIR profiles: (**a**) GAUL and (**b**) GASC.

Fourier transform infrared (FTIR) spectroscopy was utilized to ascertain the functional groups on the surfaces of raw *U. lactuca* and the synthesized GASC adsorbent. A comparative analysis of their spectra (Fig. 2a and b) revealed noticeable modifications in functional group characteristics after activation. The broad band observed at 3251.94 cm^− 1^ in *U. lactuca* corresponds to O–H stretching vibrations, whereas in GASC, this feature shifted to 3336.26 and 3206.89 cm^–1^. In the raw biomass, a strong absorption at 2927.11 cm^–1^ indicated the presence of –CH_2_ stretching groups, which appeared as broader bands at 2670.77 and 2589.15 cm^–1^ in GASC. A weak band at 1787.57 cm^–1^ in *U. lactuca* was attributed to C = O stretching of anhydride groups; in contrast, this peak shifted to 1706.59 cm^–1^ in GASC, suggesting the formation of carboxyl groups. The increased intensity of the 1706.59 cm^− 1^ band in GASC compared to the raw material indicates that sulfuric acid treatment enhanced the carbonyl (C = O) functionality^52,71^. In the spectrum of *U. lactuca* (Fig. 2a), the band at 1628.04 cm⁻¹ corresponds to C = O stretching of *β*-ketone groups. In GASC, this peak shifted to 1585.38 cm^− 1^ with higher intensity, which may be attributed to –C = C– stretching vibrations (Fig. 2b). The absorption peaks in the range of 1418.58–1251.06 cm^–1^ in *U. lactuca* indicate C–O functional groups, whereas in GASC, bands at 1373.64 and 1235.98 cm^–1^ are associated with sulfonyl (–SO) stretching vibrations. Furthermore, a broad absorption spanning 1200–850 cm^–1^ was observed in GASC, likely originating from the dehydration reaction with H_2_SO_4_, leading to the formation of –SO_3_H and –SO functionalities. These spectral changes confirm that sulfuric acid treatment transformed *U. lactuca* into GASC. Additionally, *U. lactuca* exhibited a distinct band at 1080.39 cm^–1^, assigned to –C–O–C– asymmetric stretching, while in GASC this feature was reduced and replaced by a weaker band at 769.08 cm^–1^^27,49,61,72,73^.

The N_2_ adsorption–desorption isotherm of GASC was analyzed to evaluate the influence of H_2_SO_4_ treatment on its surface properties. The specific surface area and mesopore characteristics were determined using the BET and BJH methods, respectively. The textural parameters, including average pore diameter, mesopore area, mesopore mass, total pore volume, monolayer volume, and mesopore distribution peak, are summarized in Table 2. GASC exhibited a relatively low BET surface area of 6.27 m²/g, with a monolayer volume of 1.4392 cm³ (STP)/g. The total pore volume was measured as 0.0203 cm³/g, and the mean pore size was 12.971 nm. From the adsorption analysis, the mesopore distribution peak, mesopore volume, and mesopore surface area were 1.22 nm, 0.021338 cm³/g, and 7.048 m²/g, respectively. In contrast, the desorption analysis yielded mesopore values of 1.66 nm for the distribution peak, 0.018970 cm³/g for the volume, and 4.334 m²/g for the surface area^31,49,74^.

**Table 2:** Surface area analysis of GASC adsorbent.

Method	Parameter	Value	Unit
BET analysis	Monolayer volume (*V*_m_)	1.4389	cm^3^ (STP) g^–1^
	Energy constant (the first layer) (*C*)	6.1583	
	Mean pore diameter	12.971	nm
	BET specific surface area (*a*_s, BET_)	6.2626	m^2^ g^–1^
	Total pore volume	0.020309	cm^3^ g^–1^
BJH analysis Adsorption	Pore volume (*V*_p_)	0.021338	cm^3^ g^–1^
	The pore specific surface area (*a*_p_)	7.0477	m^2^ g^–1^
	Micropore radius (cylindrical shape) (*r*_p, peak_ (Area))	1.22	nm
BJH analysis Desorption	Pore volume (*V*_p_)	0.018970	cm^3^ g^–1^
	The pore specific surface area (*a*_p_)	4.3323	m^2^ g^–1^
	Micropore radius (cylindrical shape) (*r*_p, peak_ (Area))	1.66	nm

**Fig. 3:**
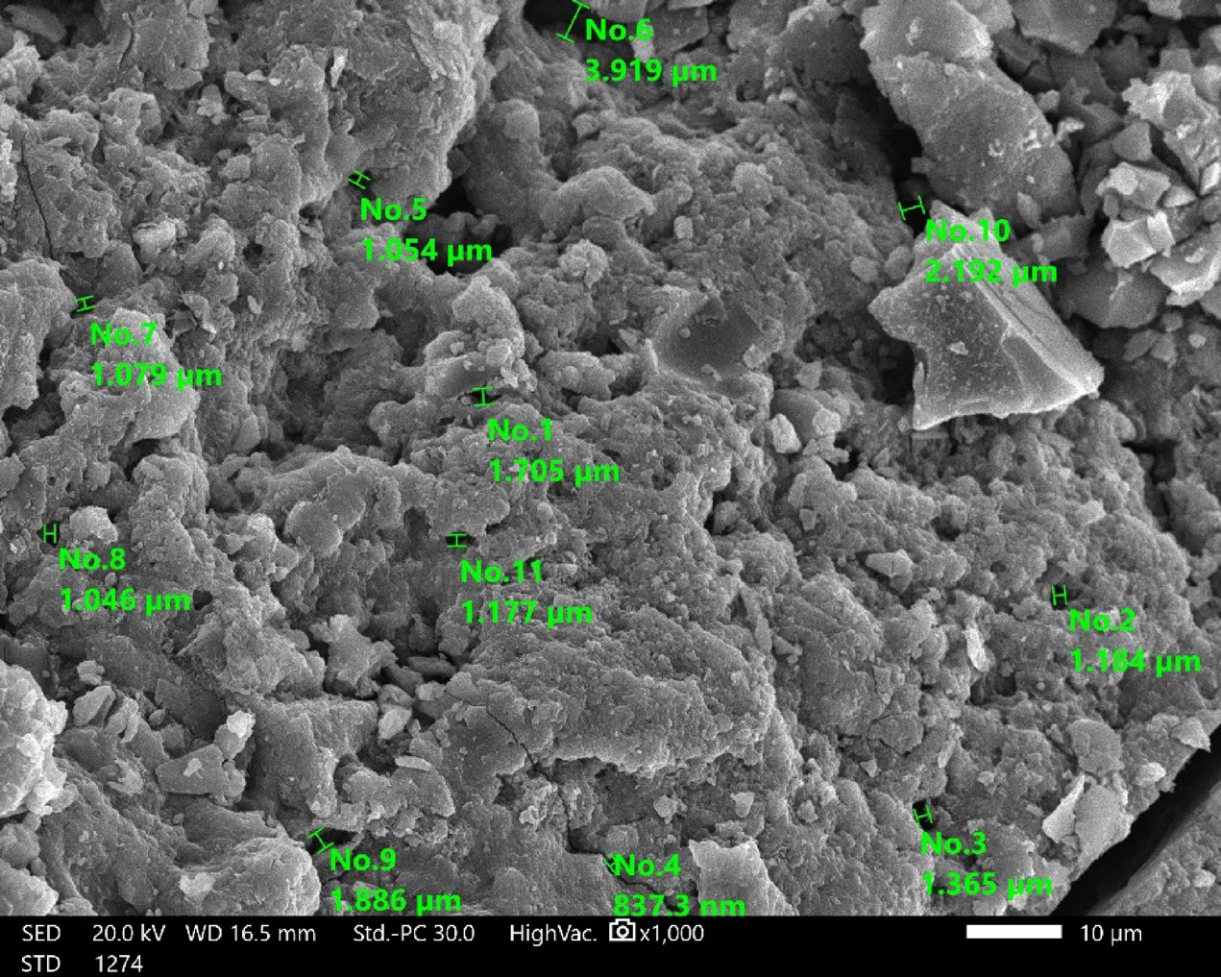
Surface morphology of GASC observed under SEM (15.0 kV, ×1000).

Figure 3 presents the SEM micrographs of GASC, confirming its structural purity and the absence of noticeable contaminants. Despite the intensive sulfuric acid treatment, the porous framework of GASC was preserved. The particle size analysis revealed a distribution ranging from 837.3 to 3919 nm, with an average particle size of 1585.85 ± 876.60 nm^49^.

**Fig. 4:**
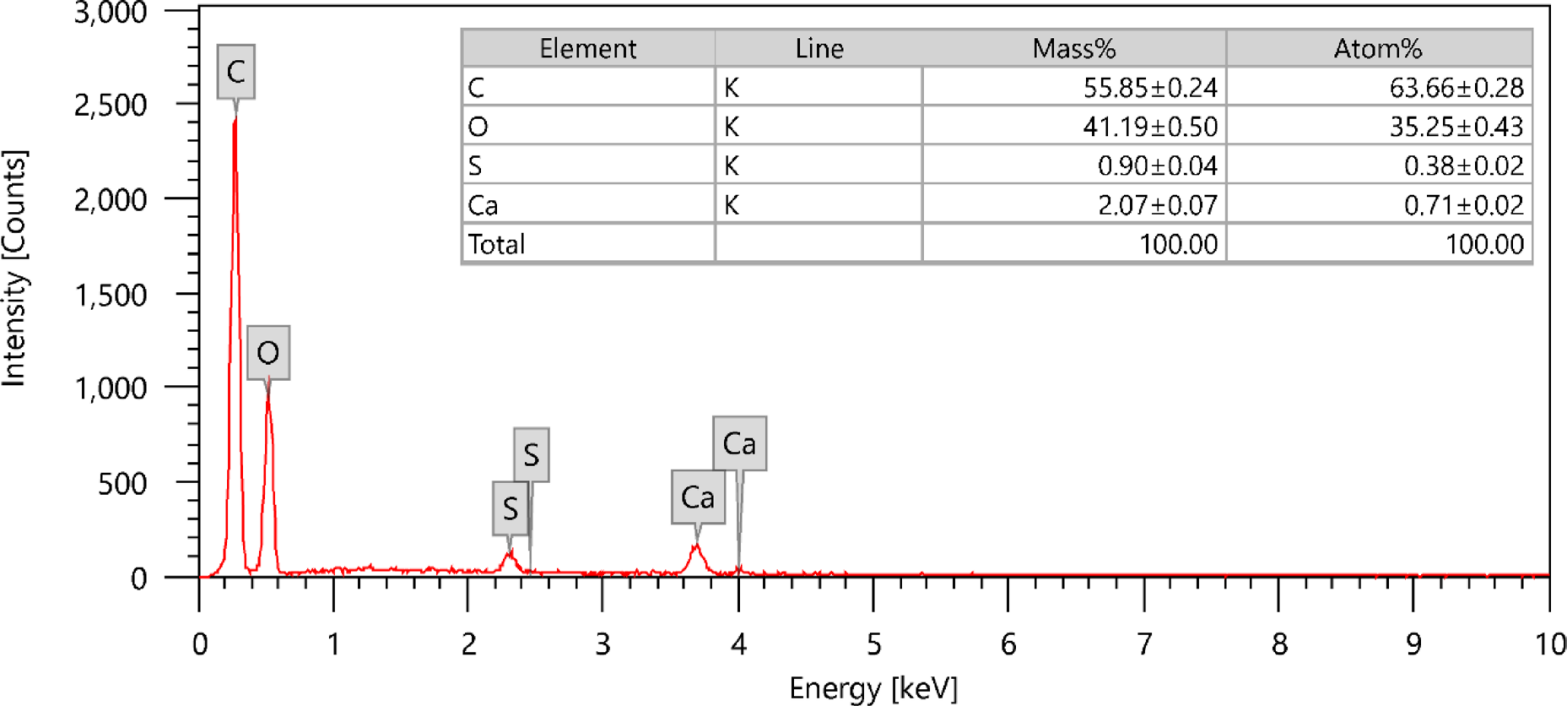
EDX analysis of GASC.

The elemental composition of the GASC adsorbent was analyzed utilizing energy-dispersive X-ray spectroscopy (EDX). As shown in Fig. 4, carbon was the dominant element, constituting 55.85 ± 0.24% of the sample. Oxygen (41.19 ± 0.50%), sulfur (0.90 ± 0.04%), and calcium (2.07 ± 0.07%) were also detected in notable proportions^49^.

**Fig. 5:**
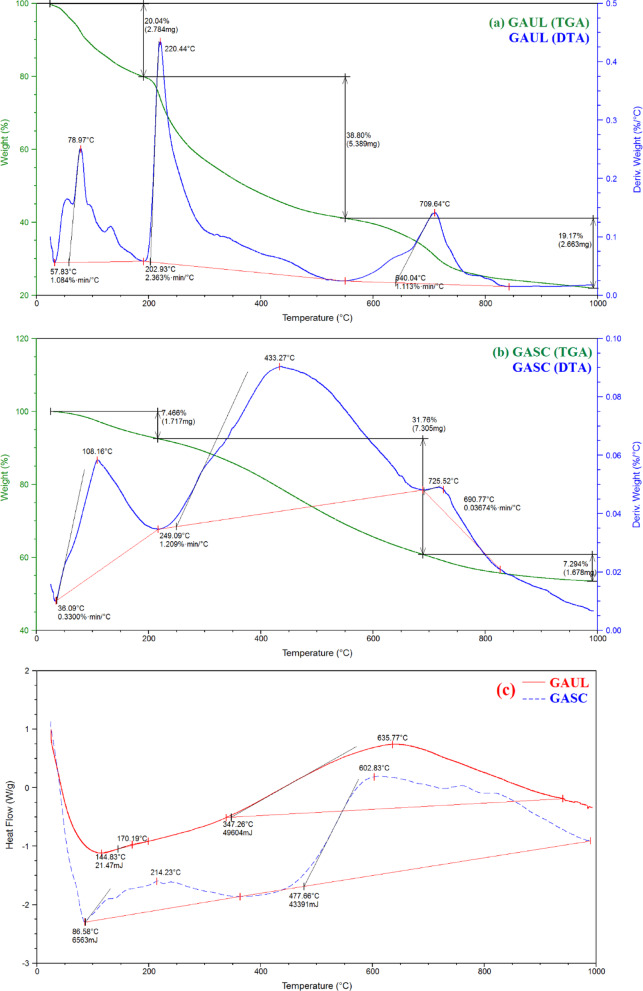
(**a**) TGA and TDA curves of GAUL; (**b**) TGA and TDA curves of GASC; (**c**) DSC thermograms of GAUL and GASC.

Thermogravimetric analysis (TGA) was employed to evaluate the impact of structural variations on the degradation behavior and thermal stability of raw GAUL and GASC (Fig. 5a and b). The samples were subjected to heating from 50 to 1000 °C in a nitrogen atmosphere. Figure 5 displays the appropriate TGA, Differential Thermal Analysis (DTA), and Differential Scanning Calorimetry (DSC) profiles. The initial mass loss observed below 200 °C was attributed to moisture evaporation in both GAUL and GASC. Beyond 200 °C, further weight reduction occurred due to the decomposition of oxygen-containing acidic functional groups. GAUL exhibited three major weight losses within the ranges of 25–200, 200–550, and 550–950 °C, with an overall mass reduction of 78.01% (Fig. 5a). Similarly, GASC also displayed three weight-loss stages between 25 and 220, 220–690, and 690–950 °C; however, its total weight loss was 46.52%, indicating higher thermal stability compared to GAUL (Fig. 5b). The convergence of TGA curves occurred at temperatures exceeding 433.27 °C for GASC and 220.44 °C for GAUL, primarily due to the degradation of carbonaceous matter^49^.

Figure 5a and b present the DTA profiles of raw GAUL and GASC. For raw GAUL, three distinct peaks were observed at 78.97, 220.44, and 709.64 °C (Fig. 5a), whereas GASC exhibited peaks at 108.16, 433.27, and 725.52 °C (Fig. 5b). The DTA results suggest that the formation of GASC from raw GAUL involved three major degradation stages associated with dehydration. Notably, the degradation bands of GASC appeared at higher temperatures following treatment with 90% H_2_SO_4_, indicating that sulfuric acid treatment significantly enhanced its thermal resistance.

DSC is a useful technique for comparing materials based on their thermal or glass transition behaviors. The DSC thermograms of raw GAUL and GASC are presented in Fig. 5c. The glass transition temperatures (*T*_g_) of GAUL occurred at 144.83 (21.47 mJ) and 347.26 °C (49604 mJ), while GASC displays *T*_g_ values at 86.58 (6563 mJ) and 477.66 °C (43391 mJ). Elevated transition temperatures promoted the formation of more crystalline structures, enhancing resistance to gelatin degradation and improving overall structural integrity^49^.

**Fig. 6:**
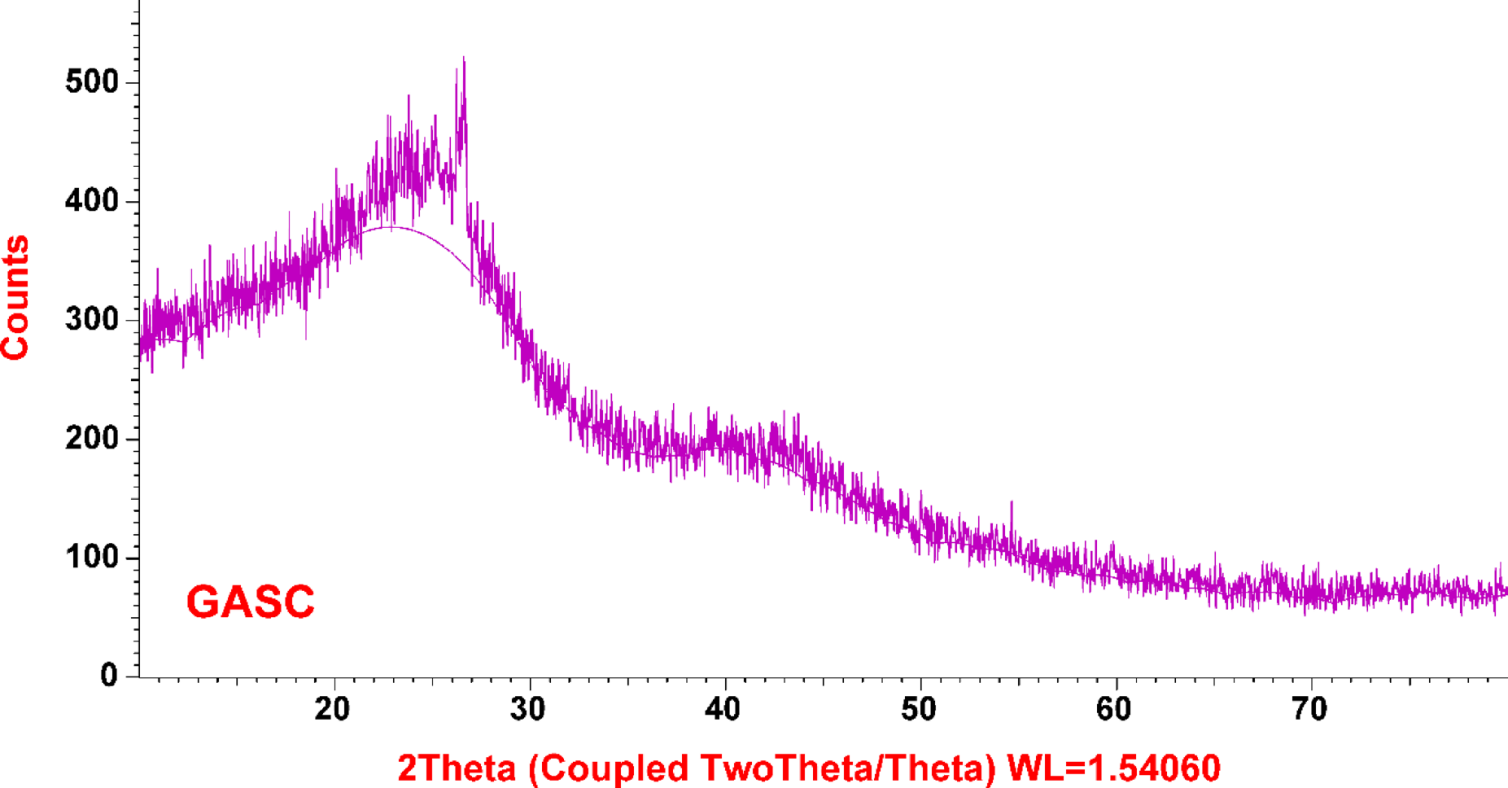
XRD analysis of synthesized GASC.

Figure 6 presents the XRD patterns of GASC, indicating an amorphous carbon structure characterized by randomly arranged aromatic layers. A broad diffraction peak around 2θ ≈ 24.9° corresponds to the C (002) plane, while a smaller peak appears at 2θ = 42.54°. Additionally, a minor peak near 2θ ≈ 15.4° (101) is associated with crystalline cellulose. This feature may also reflect the presence of inorganic components, such as quartz and albite, a plagioclase feldspar mineral^49,75–77^.

### Adsorption of AY36 dye and MR dye on GASC

#### Effect of pH

**Fig. 7:**
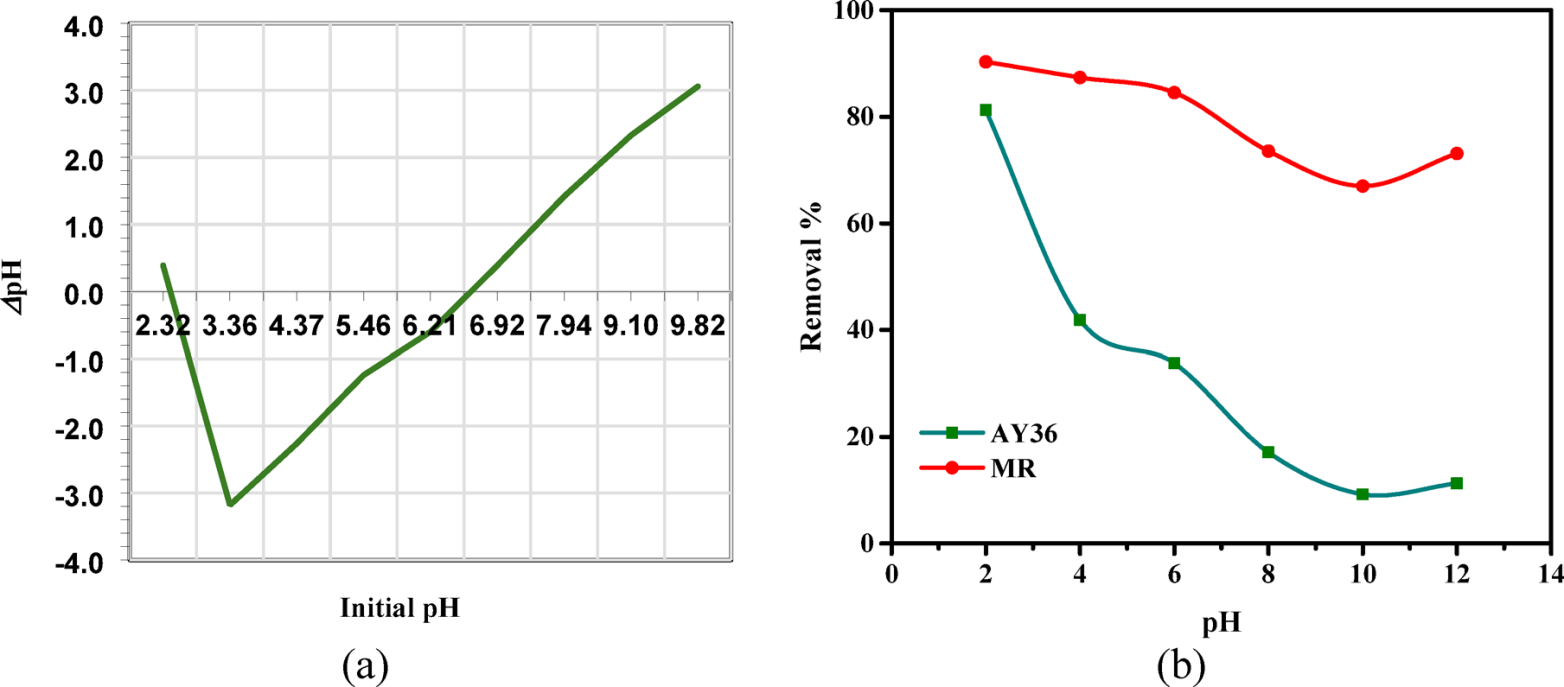
(**a**) zero point charge of GASC, and (**b**) Effect of pH on the removal efficiency of AY36 and MR dyes by GASC (initial dye concentration: 100 mg/L; adsorbent dose: 1.0 g/L; contact time: 180 min; temperature: 25 °C).

Wastewater generated by the textile industry exhibits considerable variation in pH. The adsorption process is notably influenced by the solution pH, which influences the protonation and deprotonation of functional groups, including amino, hydroxyl, and carboxyl on the biochar surface. In this study, the adsorption performance for AY36 and Methyl Red (MR) dyes was evaluated at 25 °C, using an initial dye concentration of 100 mg/L and a GASC adsorbent dose of 1 g/L. Experiments were conducted over a 180-minute period across a pH range of 2 to 12. As illustrated in Fig. 7, maximum dye removal efficiencies were observed at pH 2, achieving 90.29% for MR and 81.25% for AY36. The removal efficiencies of both dyes declined steadily as pH increased from 2 to 10, followed by a slight rise between pH 10 and 12. Several studies have highlighted the significant influence of pH on the adsorption efficiency of azo dyes. Song et al.^78^ reported a sharp decline in the adsorption capacity of Sunset Yellow as the pH increased from 2 to 4. Similarly, Khaled et al.^79^, in their study on Direct Yellow 12 removal, observed a substantial decrease in adsorption efficiency -from 98.1% to 11.1%- when the pH was elevated from 1.5 to 11.1. Eleryan et al.^21^ examined the effect of pH on Acid Yellow 11 dye removal using Mandarin Biochar-TETA (MBT), derived from *Citrus reticulata* peels, and found a reduction in removal efficiency from 66.5% to 1.3% as pH increased from 1.5 to 12. In the present study, the optimum pH for the effective removal of both AY36 and MR dyes using GASC was identified as 2.

The point of zero-charge (pH_PZC_), based on the result displayed in Fig. 7a, was calculated to be 6.95. When the solution’s pH was higher than pH_PZC_, the active sites on the biosorbent surface were negatively charged and positively charged when it was after or below the pH_PZC_^80,81^. In alkaline conditions (high pH), the abundance of hydroxide ions (OH⁻) competes with the anionic dye molecules AY36 and MR dyes for active adsorption sites, thereby reducing the overall adsorption efficiency. GASC tends to preferentially adsorb OH⁻ ions due to their high mobility and concentration, further hindering dye uptake. Conversely, at acidic pH levels, the surface of GASC becomes increasingly protonated, leading to the formation of more positively charged sites that enhance electrostatic attraction with negatively charged dye molecules. This electrostatic interaction significantly promotes the adsorption of AY36 and MR dyes, explaining the markedly higher removal efficiency observed at pH 2 (Fig. 7b). Additionally, the hydrophobic nature of the sulfonated biochar carbon contributes to this behavior. Hydrogen ions interact with the carbon surface upon contact with water, imparting a positive charge, facilitating the attraction and subsequent adsorption of anionic dye species through electrostatic forces.

#### Effect of adsorbent dosage

**Fig. 8:**
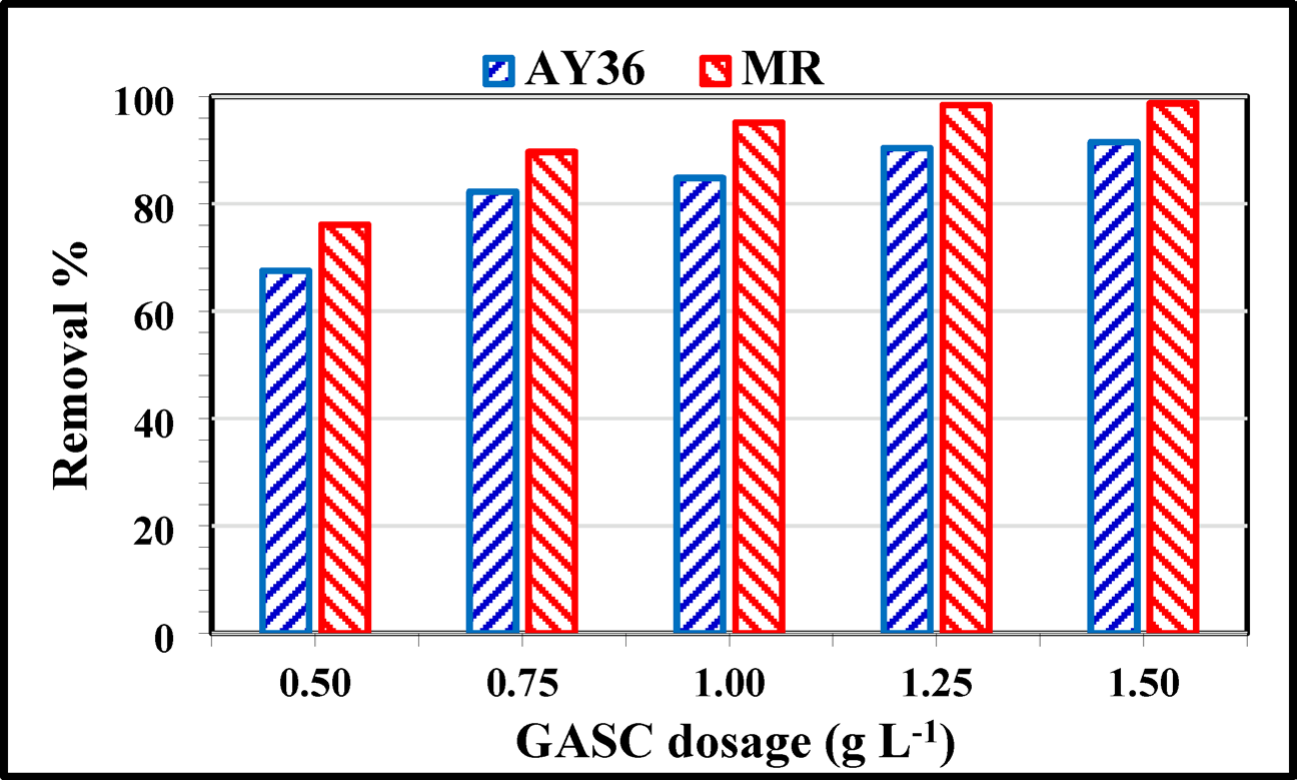
Effect of various GASC dosages on the removal% of AY36 and MR dyes (*C*_0_ of AY36 or MR dye = 100 mg/L, pH of AY36 or MR dye = 2, Time for AY36 or MR dye = 180 min, Temp. = 25 °C).

The amount of GASC used plays a pivotal role in the adsorption process, as it determines the number of available active sites for binding the target dyes at a given initial concentration. In this study, the impact of different GASC dosages on the removal of AY36 and MR dyes was examined, while all other experimental conditions were kept constant. As shown in Fig. 8, increasing the amount of adsorbent led to higher adsorption percentages for both dyes. A clear positive correlation was observed between GASC dosage and dye removal efficiency. Specifically, at an initial dye concentration of 100 mg/L, the highest removal rate for MR dye reached 98.71% with an adsorbent dose of 1.5 g/L.

Conversely, at an adsorbent dosage of 1.5 g/L, the removal efficiency of AY36 dye reached 91.46%. This elevated dye removal can be ascribed to the greater number of available adsorption sites and the expanded surface area associated with higher adsorbent concentrations. Additionally, increasing the GASC dosage while keeping the dye concentration constant provided more accessible surface area for dye molecules to interact with, thereby enhancing adsorption performance^33,35^. Based on these findings, the optimal GASC dosage for the effective removal of both AY36 and MR dyes was determined to be 1.5 g/L.

#### Effect of initial solution concentration

**Fig. 9:**
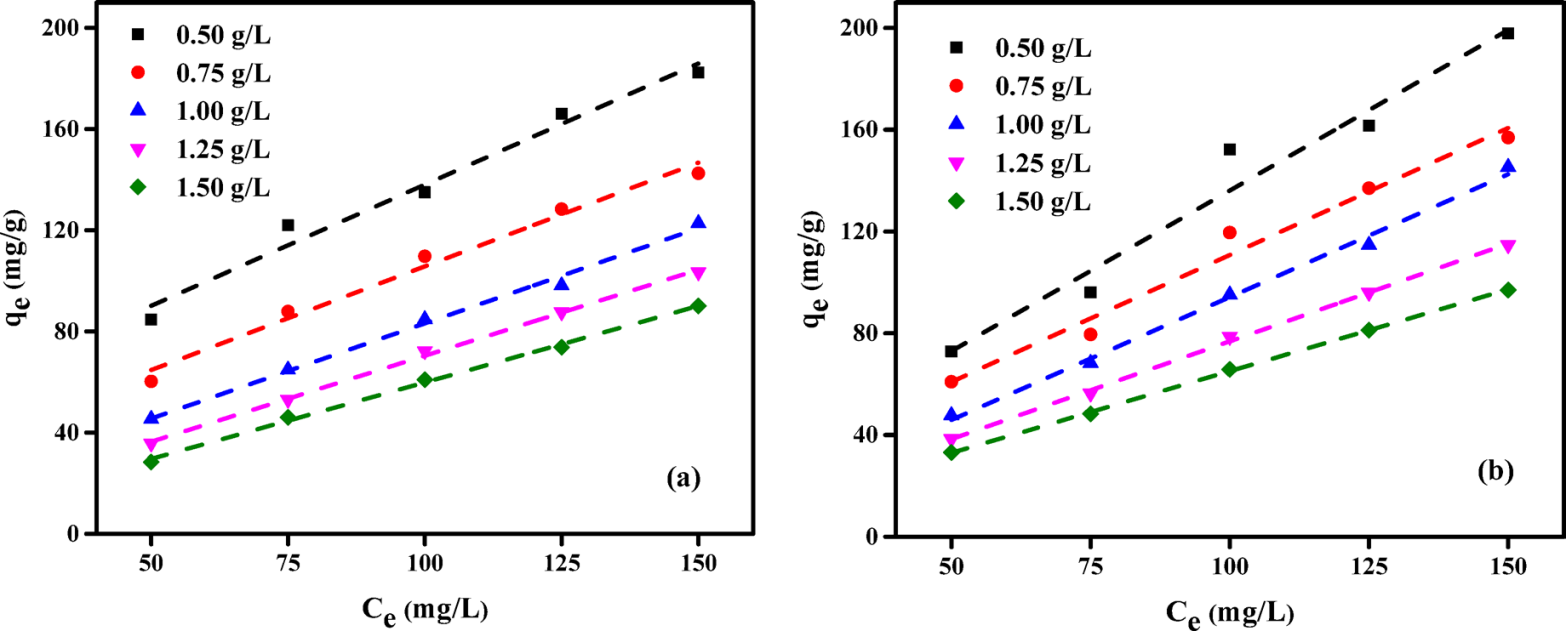
Influence of initial dye concentration on the adsorption capacity (*q*_e_) of GASC: (**a**) AY36 and (**b**) MR (contact time = 180 min, pH = 2, temperature = 25 °C).

This study explored how different initial concentrations of AY36 and Methyl Red (MR) dyes influence the adsorption capacity of GASC. Experiments were performed at a fixed contact time of 180 min and pH 2, using a range of initial dye concentrations (50, 75, 100, 125, and 150 mg/L) and GASC dosages (0.50–1.50 g/L). The corresponding results are presented in Fig. 9. A consistent increase in adsorption capacity (*Q*_e_, mg/g) was observed with rising initial dye concentrations, indicating a positive relationship. For instance, at a GASC dose of 0.50 g/L, *Q*_e_ increased from 84.72 to 182.36 mg/g as the AY36 dye concentration rose from 50 to 150 mg/L. Similarly, for GASC dosages of 0.75, 1.00, 1.25, and 1.50 g/L, the adsorption capacities ranged from 60.28 to 142.50, 45.42 to 122.78, 35.78 to 103.44, and 28.38 to 90.09 mg/g, respectively, over the same concentration range. Interestingly, as the GASC dose increased from 0.50 to 1.50 g/L at a constant initial AY36 dye concentration of 150 mg/L, the maximum adsorption capacity declined from 182.36 to 90.09 mg/g (Fig. 9a).

For MR dye, the adsorption capacity at equilibrium (*Q*_e_, mg/g) increased from 72.85 to 197.89 mg/g when the GASC dosage was fixed at 0.5 g/L and the initial dye concentration varied from 50 to 150 mg/L (Fig. 9b). Similarly, with GASC dosages of 0.75, 1.00, 1.25, and 1.50 g/L, *Q*_e_ values rose from 60.91 to 156.93, 47.71 to 145.29, 38.44 to 114.67, and 33.10 to 97.01 mg/g, respectively, across the same concentration range. However, when the GASC dosage was increased from 0.5 to 1.5 g/L while maintaining a constant initial MR dye concentration of 150 mg/L, the maximum adsorption capacity declined from 197.89 to 97.01 mg/g. Notably, GASC consistently exhibited a higher adsorption capacity for MR dye compared to AY36 dye at all tested concentrations. The results indicate that the adsorption performance is closely associated with the initial dye concentration in solution. The observed trends can be attributed to the limited number of active sites on the adsorbent surface, which may be insufficient to accommodate high dye concentrations. At lower concentrations, solute molecules have greater access to available adsorption sites, resulting in more efficient uptake.

#### Effect of contact time using GASC

**Fig. 10:**
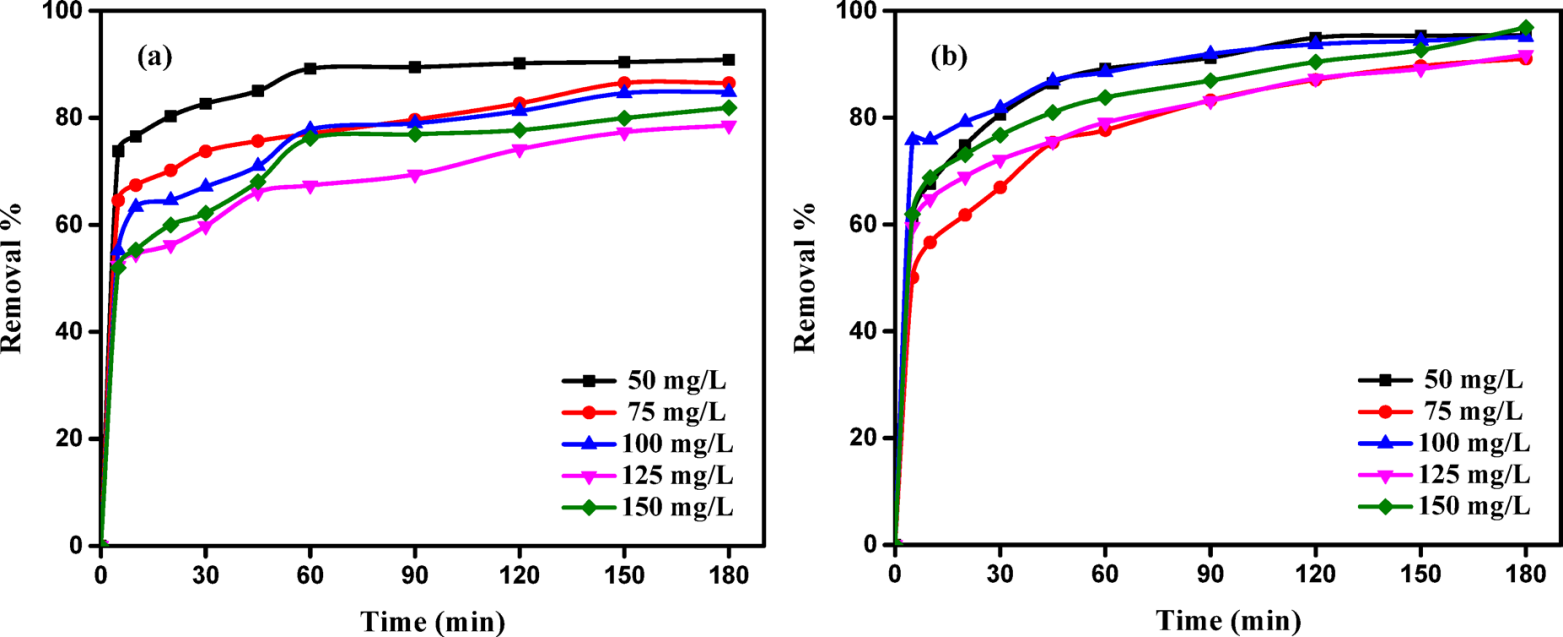
Influence of contact time on the adsorption of (**a**) AY36 and (**b**) MR dyes by GASC (adsorbent dose: 1.0 g/L, time: 180 min, pH 2, 25 °C).

Contact time is a fundamental parameter influencing all mass transfer processes, including adsorption. The effect of contact time on the removal efficiencies of AY36 and MR dyes by GASC over a 180-minute period is presented in Fig. 10. As shown, the equilibrium time is dependent on the type and initial concentration of the dye, with variations observed between different adsorbents. In Fig. 10a, it is evident that a substantial portion of AY36 dye is adsorbed within the first 5 min. At an initial concentration of 50 mg/L, the removal efficiency reached 73.75%, whereas it was 51.94% for a starting concentration of 150 mg/L. Subsequently, dye removal increased significantly up to 60 min, followed by a gradual rise with a diminishing rate until equilibrium was approached around the 150th minute.

A similar trend was observed for MR dye, as depicted in Fig. 10b. Within the initial 5 min, a steady increase in MR dye removal was recorded, with removal efficiencies of 61.33% and 61.93% at initial concentrations of 50 mg/L and 150 mg/L, respectively. This was followed by a continued rise in removal efficiency up to the 180-minute mark. At lower dye concentrations, the higher ratio of available surface area to dye molecules led to reduced dependence of the adsorption process on initial concentration. In contrast, at higher concentrations, this ratio declined, resulting in a greater influence of initial concentration on removal performance. These findings indicate that the extent of dye removal is closely related to the initial dye concentration, consistent with previous reports^20,82^.

### Adsorption isotherm studies

**Fig. 11:**
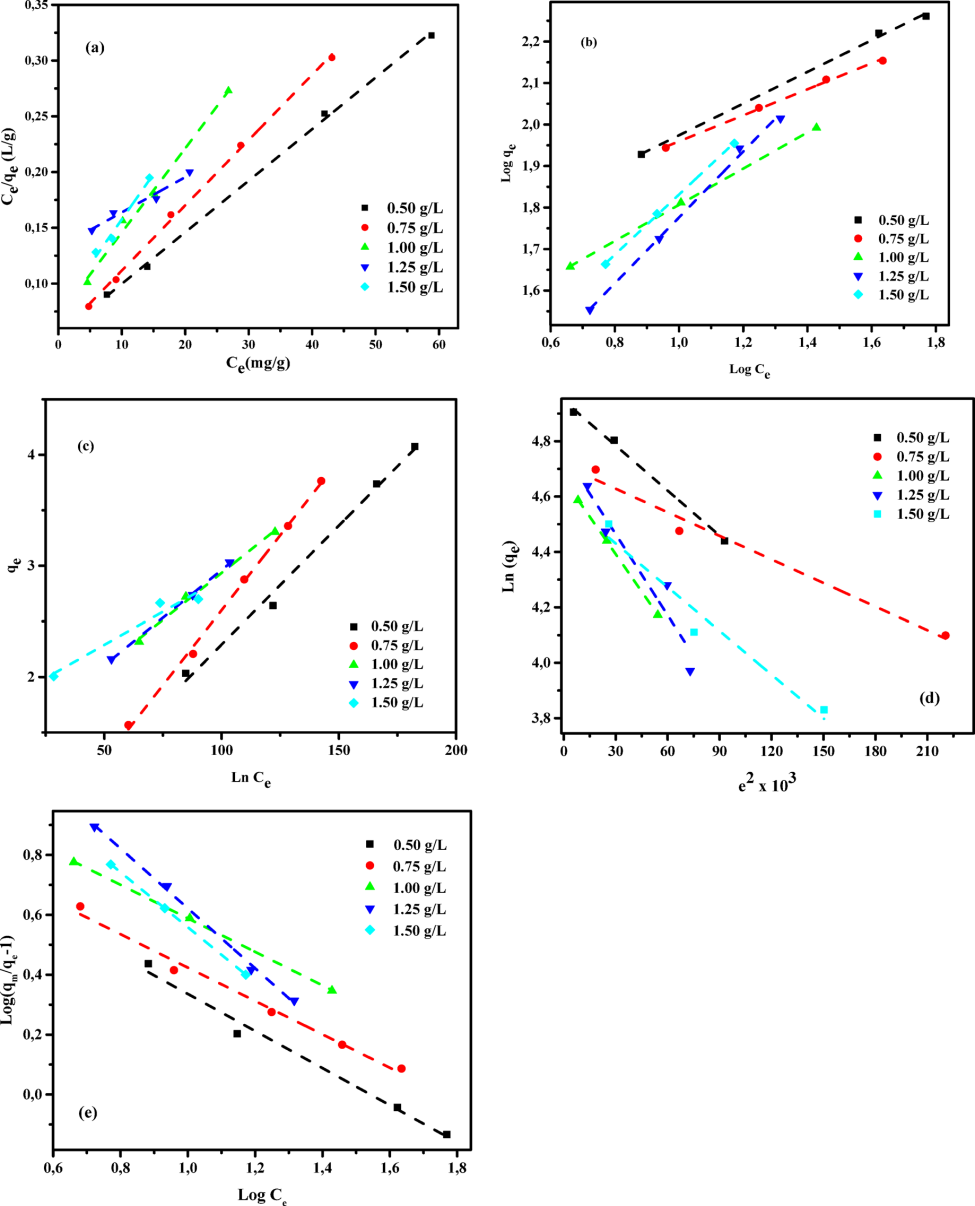
Isotherm model profiles -(**a**) LIM, (**b**) FIM, (**c**) TIM, (**d**) DRIM, and (**e**) GIM- for AY36 dye adsorption onto GASC (*C₀* = 50–150 mg/L; adsorbent dose = 0.5–1.5 g/L; T = 25 °C; time = 180 min).

**Fig. 12:**
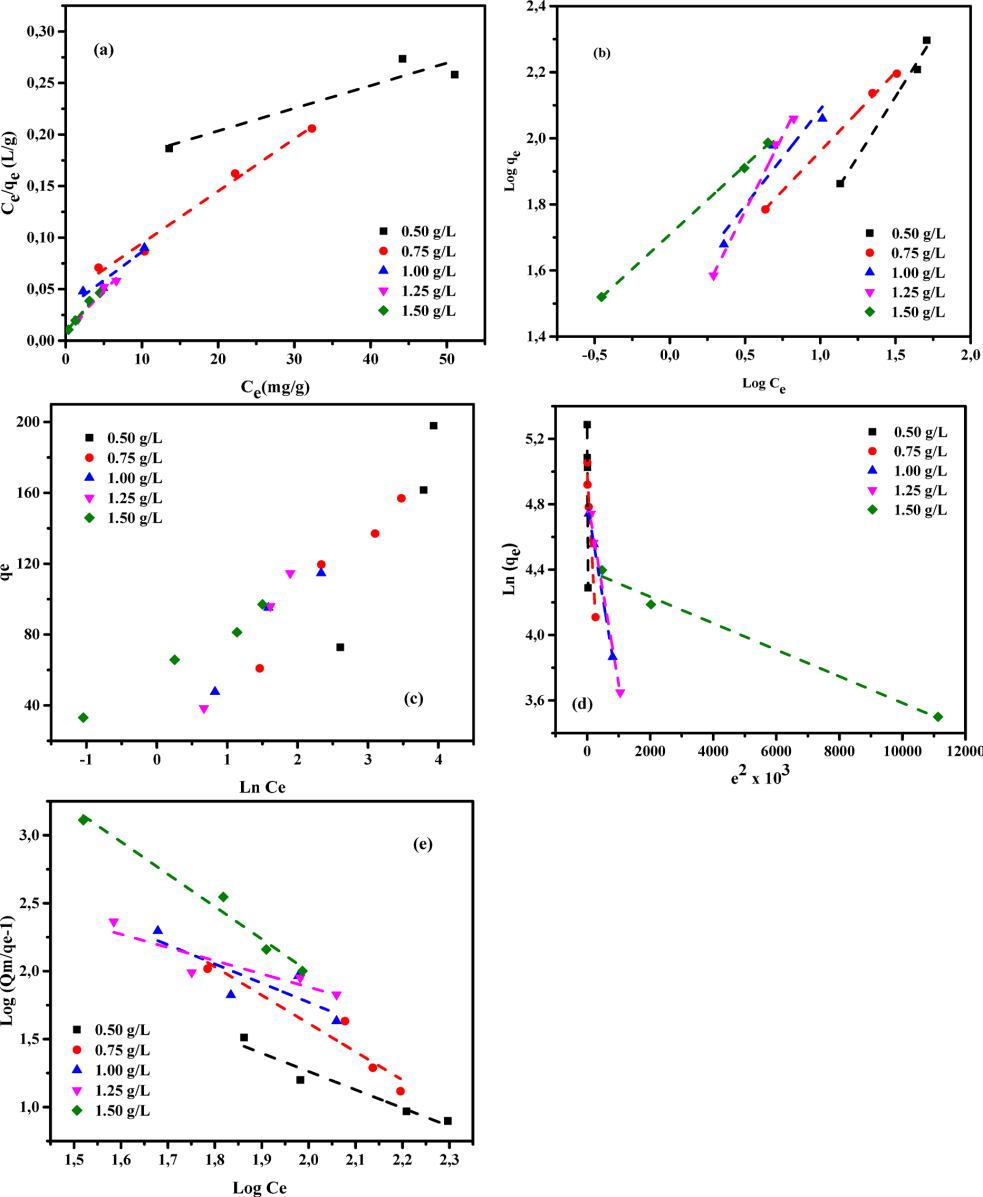
Isotherm fitting curves of MR dye adsorption onto GASC: (**a**) LIM, (**b**) FIM, (**c**) TIM, (**d**) DRIM, and (**e**) GIM (*C₀* = 50–150 mg/L; adsorbent dose = 0.5–1.5 g/L; T = 25 °C; contact time = 180 min).

Adsorption isotherms serve as essential analytical models for understanding the mechanisms governing the transfer of substances from aqueous phases or porous media to solid surfaces under constant conditions^83,84^. Equilibrium in the adsorption process is reached when the amount of adsorbate accumulated on the solid surface balances the amount remaining in the solution. This equilibrium state occurs after sufficient contact time between the adsorbent and adsorbate, during which the concentration of the pollutant in the bulk solution stabilizes and aligns with that at the solid–liquid interface^85,86^. The mathematical relationship between the concentration of a solute remaining in the liquid phase and the amount adsorbed onto the solid phase -originally formulated and validated by Shoaib et al. (2025)^87^- remains a foundational tool in adsorption studies. This correlation plays a critical role in the theoretical modelling, optimization, and real-world application of pollutant removal processes. As noted by Chen et al. (2024)^29^, analyzing thermodynamic principles and physicochemical interactions enables a deeper understanding of adsorption behavior, including surface properties and the affinity of adsorbents for specific contaminants. To analyze the experimental data, various adsorption isotherm models have been employed, including Langmuir (LIM), Freundlich (FIM), Temkin (TIM), Dubinin–Radushkevich (DRIM), and Generalized Isotherm (GIM) models^37,88,89^. The performance of these six isotherms in describing the adsorption behavior of AY36 and MR dyes onto GASC was evaluated, with the corresponding results summarized in Tables 3 and 4 and illustrated in Figs. 11 and 12.

#### Langmuir isotherm

Originally proposed by Langmuir in 1916^90^, the Langmuir isotherm model (LIM) describes the adsorption of a solute from a liquid phase onto a uniform surface, assuming monolayer coverage without lateral movement of adsorbate molecules across the surface plane. This model presumes a finite number of identical and energetically equivalent adsorption sites^38^. LIM was employed in this study to estimate the maximum adsorption capacity (*Q*_*m*_, mg/g) corresponding to complete monolayer formation on the adsorbent. The mathematical expression of the LIM is provided in Eq. 3.

3 $$\:\frac{{C}_{e}}{{q}_{e}}=\frac{1}{{K}_{L}}{Q}_{m}+\frac{1}{{Q}_{m}}x{C}_{e}$$

where *Ce* (mg/L) represents the concentration of adsorbate in solution at equilibrium, *q*_e_ (mg/g) represents the adsorption capacity at equilibrium, *K*_L_ (L/mg) is the LIM adsorption constant, and *Q*_m_ (mg/g) is the maximum adsorption capacity at monolayer coverage.

LIM-related data are summarized in Tables 3 and 4, while the corresponding LIM plots for AY36 and MR dyes are illustrated in Figs. 11(a) and 12(a), respectively. In this study, the GASC adsorbent exhibited strong linear correlations with the Langmuir isotherm model, as indicated by R² values ranging from 0.951 to 0.998 for AY36 and from 0.887 to 0.988 for MR, highlighting its effective adsorption potential for both dyes. The calculated monolayer adsorption capacities (*Q*_*m*_) were 216.45 mg/g for AY36 and 454.55 mg/g for MR. The slope and intercept derived from the linear plots of *C*_*e*_/*q*_*e*_ versus *C*_*e*_, illustrated in Figs. 11(a) and 12(a), were used to determine the Langmuir constants 1/*Q*_*m*_ and 1/*Q*_*m*_*K*_*L*_. The *K*_*L*_ values ranged from 0.0091 to 0.042 L/mg for AY36 dye and from 0.00094 to 0.0726 L/mg for MR dye, confirming favorable interactions between the adsorbate molecules and the GASC surface. Overall, these results suggest that the Langmuir model appropriately describes the adsorption behavior of AY36 dye on GASC, whereas its applicability to MR adsorption is comparatively limited. Detailed characterization indicated that both dyes were predominantly adsorbed in a monolayer on the GASC surface.

#### Freundlich isotherm

The Freundlich isotherm model (FIM)^91^ was the first equation applied to analyze the adsorption behavior in this study. Owing to its empirical and exponential nature, the FIM suggests that the concentration of adsorbate on the surface of the adsorbent rises proportionally with the increase in adsorbate concentration in the solution. The corresponding mathematical expression is provided in Eq. (4).

4 $$\:Ln\:{q}_{e=\:}\text{ln}{K}_{F}+\frac{1}{n}\text{ln}{C}_{e}$$

where *n* and *K*_F_ (mg^1 − 1/n^ g^− 1^ L^1/n^) denote the Freundlich constants, representing the intensity and capacity of adsorption, respectively.

Tables 3 and 4 present the parameters obtained from the Freundlich isotherm model (FIM), while Figs. 11(b) and 12(b) illustrate the corresponding FIM plots for AY36 and MR dyes, respectively. From the linear plots of ln(*q*_*e*_) versus ln(*C*_*e*_), the intercept and slope shown in Figs. 11(b) and 12(b) were used to calculate the logarithmic values of the Freundlich constants *K*_*F*_ and *1/n*. The parameter *K*_*F*_ (L/g) reflects the binding strength between the adsorbent (GASC) and the dyes (AY36 and MR) at equilibrium. As noted by Fan et al. (2012)^92^, a *1/n* value below 1 indicates favorable adsorption and a stronger affinity between the adsorbate and adsorbent. In this study, all *1/n* values listed in Tables 3 and 4 are less than one, suggesting that the adsorption of both dyes onto GASC is efficient and occurs predominantly through physical interactions. The linear relationship between log(*q*_*e*_) and log(*C*_*e*_) was used to determine the FIM correlation coefficients, which are also shown in Figs. 11(b) and 12(b). For both AY36 and MR dyes, the GASC adsorbent demonstrated higher correlation coefficients with the Freundlich model (*R*^2^ = 0.995–1.000 for AY36 dye and *R*^2^ = 0.903–1.000 for MR dye) than with the Langmuir model, indicating a better fit.

#### Temkin isotherm

The Temkin isotherm model (TIM)^93^ describes adsorption based on the assumption of indirect interactions between adsorbate and adsorbent molecules. According to this theory, the heat of adsorption decreases linearly with increasing surface coverage due to adsorbate–adsorbent interactions. The model further assumes a uniform distribution of binding energies up to a certain maximum value. The Temkin isotherm can be mathematically represented by Eq. (5), which is often expressed in a simplified linear form as shown in Eq. (6)^38^.

5 $$\:{Q}_{e}=\frac{RT}{{B}_{T}}\text{l}\text{n}\left({A}_{T}{C}_{e}\right)$$

6 $$\:{Q}_{e}=\frac{RT}{{B}_{T}}\text{ln}\left({A}_{T}\right)+\frac{RT}{{B}_{T}}\text{l}\text{n}\left({C}_{e}\right)$$

where *A*_T_ (L/mg) is thr Temkin isotherm constant, *B*_T_ (J g mol^–1^ mg^–1^) represents the adsorption energy (heat of adsorption) variation factor, *R* (8.314 J mol^–1^ K^–1^) represents the universal gas constant, and *T* (K) represents the absolute temperature.

The Temkin isotherm model (TIM) constants, *A*_*T*_ and *B*_*T*_, were derived from the linear relationship between *q*_*e*_ and ln*C*_*e*_, as illustrated in Figs. 11(c) and 12(c). In these plots, the slope corresponds to *A*_*T*_ (g/L), while the intercept indicates the value of *B*_*T*_. Tables 3 and 4 provide a comprehensive summary of the TIM parameters. The calculated correlation coefficients -ranging from *R*^2^ = 0.908–0.999 for AY36 dye and *R*^2^ = 0.947–0.999 for MR dye - demonstrate a strong agreement with the experimental data across varying temperatures, confirming the suitability of the TIM for describing the adsorption process. The parameter *B*_*T*_, which reflects the heat of adsorption and represents the energy released during the adsorbate–adsorbent interaction, is critical in evaluating the adsorption mechanism. Based on the obtained *B*_*T*_ values, the adsorption of AY36 and MR dyes onto the GASC surface occurred at relatively low adsorption energies, indicating that the process predominantly follows a physisorption mechanism.

#### The dubinin–radushkevich (D-R) isotherm

The Dubinin–Radushkevich isotherm model (DRIM) is commonly employed to assess the apparent free energy associated with porosity and adsorption behavior^31,94^. Unlike models that assume surface homogeneity or uniform adsorption potential, DRIM is based on a more general approach that does not require such assumptions. The standard form of the DRIM is provided in Eq. (7), while its linearized version is illustrated in Eq. (8).

7 $$\:{q}_{e}={Q}_{m}\text{e}\text{x}\text{p}(-K{\epsilon\:}^{2})$$

8 $$\:ln{q}_{e}=ln{Q}_{m}-{K\epsilon\:}^{2}$$

where *Q*_*m*_ (mg/g) denotes the theoretical saturation capacity, *K* (mol^2^ (kJ^2^)^–1^) denotes a constant related to the adsorption energy, and ԑ represents the Polanyi potential, which can be determined using Eq. (9):

9 $$\epsilon =\text{R}\text{T}\times\:\text{l}\text{n}(1+\frac{1}{{\text{C}}_{e}})$$

In the linear form of the Dubinin–Radushkevich isotherm, the slope of the *q*_*e*_ versus *ε²* graph represents the constant *K* (mol^2^ kJ⁻^2^), while the intercept yields the maximum adsorption capacity, *Q*_*m*_ (mg/g). The mean free energy of adsorption (*E*), which reflects the energy change associated with transferring one mole of solute from an infinite distance in the solution phase to the surface of the adsorbent, was calculated using the *K* value as expressed in Eq. (10).

10 $$\:E=1/\sqrt{\left(2K\right)}$$

The Dubinin–Radushkevich isotherm model (DRIM) was employed to evaluate the equilibrium data and determine whether the adsorption of AY36 and MR dyes onto the GASC adsorbent is governed by physical or chemical mechanisms. According to the Polanyi potential theory underlying this model, the adsorption process continues until the pore structure of the adsorbent reaches saturation. Tables 3 and 4 summarize the DRIM constants and correlation coefficients obtained from experiments conducted at different adsorbent dosages. The mean adsorption energy (*E*) serves as a key parameter for identifying the adsorption mechanism. Specifically, the nature of adsorption can be inferred from *E* values as follows: *E* < 8 kJ/mol suggests physical adsorption, values between 8 and 16 kJ/mol indicate ion exchange, and *E* > 16 kJ/mol corresponds to chemical adsorption^31,94^. An analysis of the estimated binding energy (*E*) values listed in Tables 3 and 4 reveals that, for all tested dosages of the GASC adsorbent, *E* remains below 8 kJ/mol for both AY36 and MR dyes. This indicates that the adsorption mechanism for both dyes is predominantly physical in nature. The correlation coefficients (*R*^2^) obtained from the Dubinin–Radushkevich isotherm model range from 0.884 to 1.000 for AY36 dye and from 0.975 to 1.000 for MR dye across the various adsorbent dosages, demonstrating good agreement with the experimental data (Figs. 11(d) and 12(d); Tables 3 and 4). Additionally, the GASC adsorbent exhibited maximum monolayer adsorption capacities (*Q*_*m*_) of 140.70 mg/g for AY36 dye and 193.37 mg/g for MR dye. These results suggest that while DRIM provides a limited fit for the AY36 dye adsorption data, particularly when compared to the FIM and TIM models, it offers a more suitable representation for MR dye adsorption, alongside the Freundlich model.

#### Generalized isotherm equation

The Generalized isotherm equation is given in the linear form by Eq. (11):

11 $$\:log\left[\frac{{Q}_{\text{m}}}{{q}_{\text{e}}}-1\right]=log{K}_{\text{G}}-{N}_{\text{b}}log{C}_{\text{e}}$$

where *N*_b_ is the cooperative binding constant, *K*_G_ (mg/L) is the saturation constant, and *Q*_m_ (mg/g) represents the maximum adsorption capacity of the adsorbent (as determined from LIM). *C*_*e*_ (mg/L) and *q*_*e*_ (mg/g) represent the equilibrium concentrations of AY36 or MR dye in the liquid and solid phases, respectively. The plots in Figs. 11(e) and 12(e) illustrate the linear relationship between log[*(Q*_*m*_*/q*_*e*_*) – 1*] and log *C*_*e*_, where the slope and intercept correspond to the *N*_*b*_ and log *K*_*G*_ constants, respectively. The isotherm parameters were determined through linear regression analysis, and the corresponding correlation coefficients (R^2^) were calculated. Tables S1 and S2 summarize the estimated parameters along with their associated R^2^ values.

### Analysis of error functions for the optimal isotherm model

To identify the most appropriate isotherm model for describing the adsorption of AY36 and MR dyes onto GASC, the correlation coefficients (*R*²) derived from the Langmuir (LIM), Freundlich (FIM), Temkin (TIM), Dubinin–Radushkevich (DRIM), and Generalized Isotherm (GIM) models were evaluated against the experimental equilibrium data. In addition to *R*², the optimal model fit was further assessed using a range of error functions. These included average percent error (APE), sum of squared errors (ERRSQ), chi-square error (χ²), hybrid error function (HYBRID), Marquardt’s percent standard deviation (MPSD), sum of absolute errors (EABS), and root mean square error (RMS), all of which quantify the deviation between experimental results and model predictions^95^. The comparative analysis of error functions -used to evaluate the agreement between experimental data for GASC and values predicted by theoretical isotherm models- is presented in Table 3. According to the data in Table 3, the Freundlich isotherm model exhibited the lowest values for all considered error metrics (APE, χ², RMS, ERRSQ, HYBRID, EABS, and MPSD) in the case of AY36 dye, indicating its superior fit. For MR dye, the Temkin model provided the best performance across these error functions. Based on correlation coefficients, the Freundlich (FIM), Temkin (TIM), and Generalized Isotherm (GIM) models demonstrated strong agreement with experimental data for AY36 dye, with FIM emerging as the most appropriate when error functions were considered. In contrast, for MR dye, the Freundlich and Dubinin–Radushkevich (DRIM) models showed the highest correlation coefficients, whereas the Temkin model achieved the best fit based on error function analysis^55^.

**Table 3:** Error function values of isotherm models applied to the equilibrium adsorption of AY36 and MR dyes on GASC.

Dye	Isotherm Model	APE (%)	X^2^	Hybrid	ERRSQ	MPSD	EABS	RMS
AY36	Linear LIM	3.44	1082.14	6011.92	123723.37	16.21	1573.04	15.38
	Non-Linear LIM	0.43	15.10	83.88	1531.66	2.03	175.02	1.93
	FIM	0.001	0.0001	0.001	0.007	0.006	0.346	0.005
	TIM	1.17	79.79	498.66	7945.75	5.27	378.18	4.97
	DRIM	4.94	849.03	6064.47	72214.89	21.11	1074.91	19.75
	GIM	4.83	1490.60	8768.26	138808.87	22.26	1623.99	21.06
	RPM	0.03	0.17	0.77	15.45	0.18	19.66	0.17
MR	Linear LIM	1.91	284.06	1775.35	38113.63	8.57	828.29	8.08
	Non-Linear LIM	0.11	1.18	6.55	142.39	0.52	53.36	0.49
	FIM	2.43	319.78	2459.83	51487.94	10.09	878.82	9.40
	TIM	0.001	0.00002	0.0001	0.001	0.002	0.159	0.002
	DRIM	2.12	277.46	1981.83	41881.54	9.06	818.60	8.47
	GIM	1.90	39.89	234.67	640.03	8.77	110.28	8.30

### Adsorption kinetic studies

**Fig. 13:**
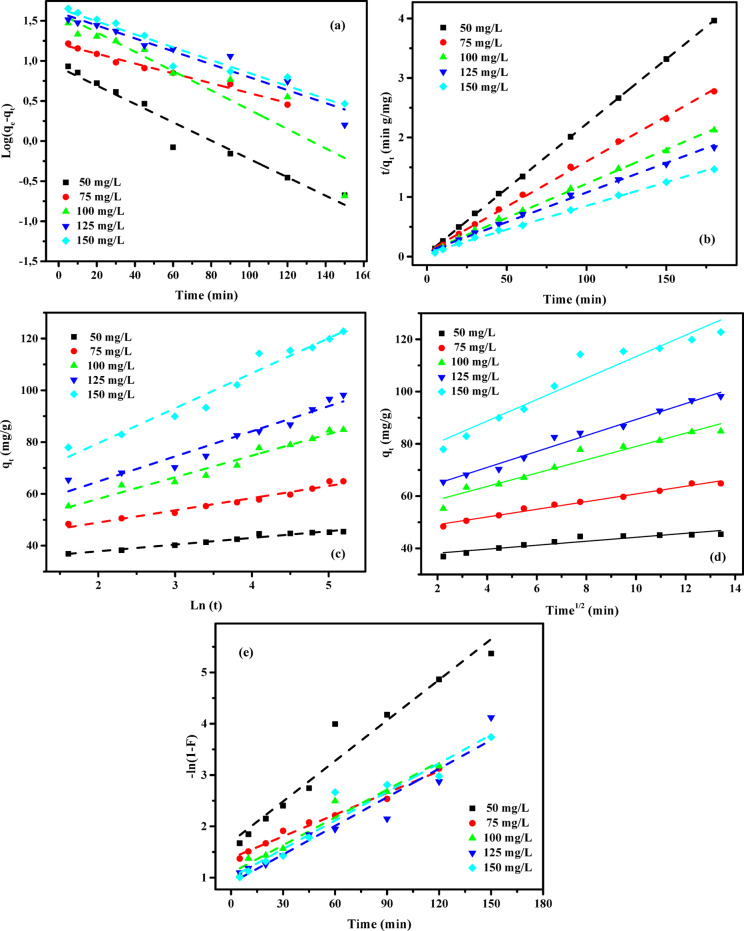
The plot of (**a**) PFOM, (**b**) PSOM, (**c**) EM, (**d**) IPDM, and (**e**) FDM of adsorption of AY36 dye by GASC adsorbent (*C*_0,_ dye = (50–150 mg/L), *C*_*0*_, GASC = (1.0 g/L), Temp. = 25 °C).

**Fig. 14:**
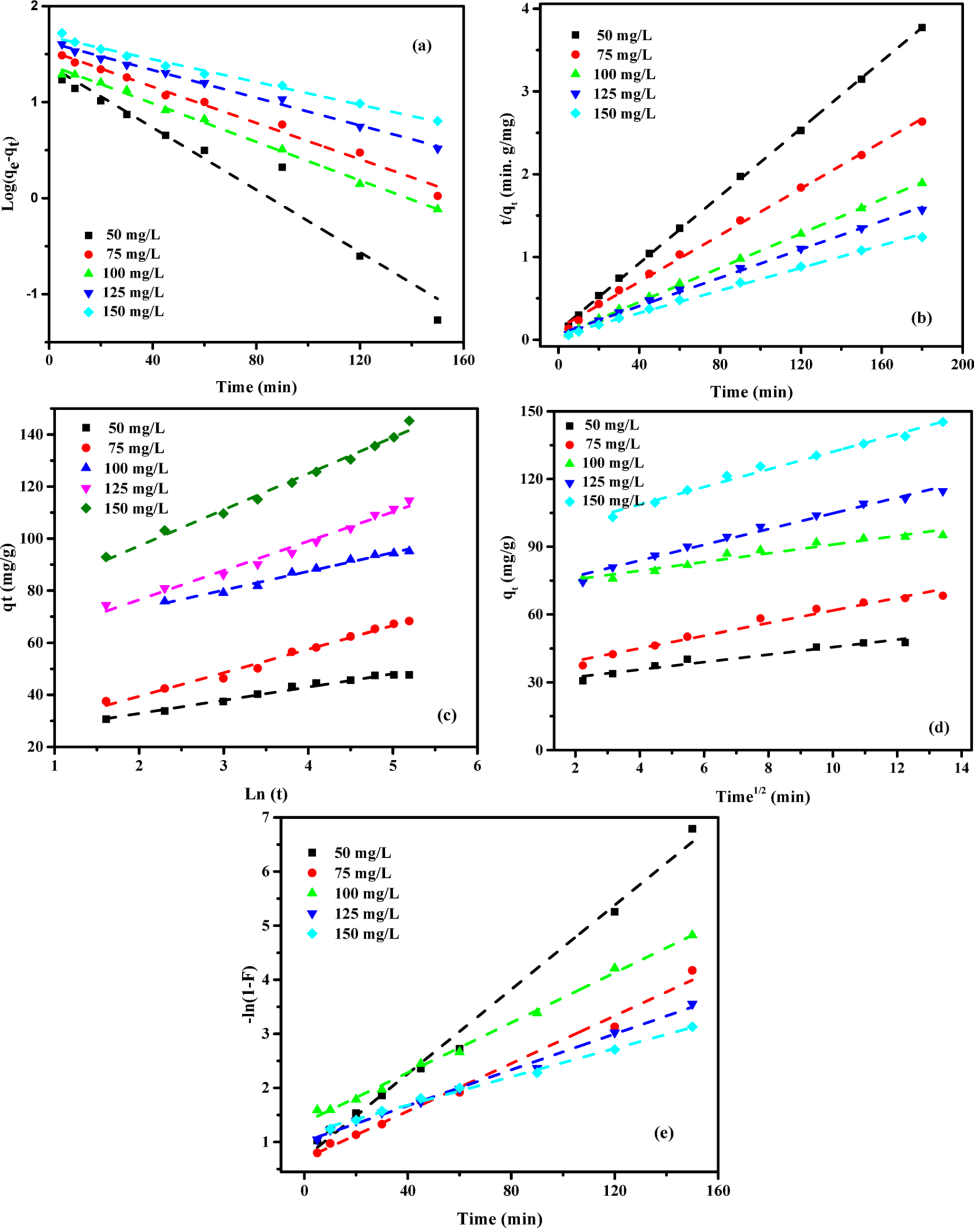
The plot of (**a**) PFOM, (**b**) PSOM, (**c**) EM, (**d**) IPDM, and (**e**) FDM of adsorption of MR dye by GASC adsorbent (*C*_0,_ MR dye = (50–150 mg/L), *C*_0_, GASC = (1.0 g/L), Temp. = 25 °C).

 To investigate the adsorption kinetics of AY36 and MR dyes onto GASC, an extensive analysis incorporating various models -covering diffusion control, mass transfer, and chemical reaction mechanisms- was conducted. The primary goal was to identify the most suitable operational conditions for potential scale-up in batch production^55^. A thorough understanding of kinetic parameters is essential for predicting adsorption rates and plays a critical role in the design, optimization, and simulation of adsorption systems. Accordingly, several kinetic models were applied, including the pseudo-first-order (PFOM)^96^, pseudo-second-order (PSOM)^97^, Elovich model (EM)^85^, Intraparticle diffusion model (IDM)^38^, and film diffusion model (FDM)^35^, to evaluate the removal efficiency of AY36 and MR dyes using GASC. The goodness of fit between the model predictions and experimental data was determined using the correlation coefficient (*R*^2^), with the results presented in Figs. 13 and 14, and detailed in Tables S3-S6.

#### Pseudo-first-order model (PFOM)

The adsorption rate constant was determined using the theoretical basis of the Lagergren pseudo-first-order model (PFOM)^98^, which serves as an initial approach for describing adsorption kinetics based on the adsorption capacity. The PFOM kinetic expression is provided in Eq. (12).

12 $$\:\text{log}\left({q}_{\text{e}}-{q}_{\text{t}}\right)=\text{log}\left({q}_{\text{e}}\right)-\frac{{k}_{1}}{2.303}t$$

The variables *q*_t_ and *q*_e_ (mg/g) denote the volumetric quantities of ions adsorbed at time *t* and equilibrium, respectively. The parameter *k*_*1*_ (min^− 1^) denotes the rate constant for the adsorption process described by the pseudo-first-order model (PFOM). The values of *k*_*1*_ and *q*_*e*_ were determined from the slope and intercept of the linearized plots of log(*q*_*e*_*−q*_*t*_) vs. (*t*), as shown in Figs. 13(a) and 14(a). As presented in Tables S3 and S5, the kinetic model’s correlation coefficients (*R*^2^) range between 0 and 1, with values closer to unity indicating a better model fit. The consistently high *R*^2^ values, all exceeding 0.850, reflect a strong agreement between the predicted and experimental adsorption capacities (*q*_*e*_). These results affirm that the PFOM effectively describes the adsorption behavior of both AY36 and MR dyes onto GASC. Furthermore, data in Tables S3 and S5 show no clear pattern of increasing or decreasing R^2^ values with rising GASC concentrations in the 0.5–1.5 g/L range for either dye.

#### Pseudo-second-order model (PSOM)

In this study, the pseudo-second-order model (PSOM) was applied to assess the adsorption efficiency of GASC in removing AY36 and MR dyes. According to the PSOM framework, the adsorption rate is assumed to be proportional to the square of the number of unoccupied active sites on the adsorbent. Additionally, the quantity of solute adsorbed on the surface significantly influences the overall reaction kinetics. The mathematical formulation of the PSOM is presented in Eq. (13).

13 $$\:\left(\frac{t}{{q}_{t}}\right)=\frac{1}{{k}_{2}{q}_{e}^{2}}+\frac{1}{{q}_{e}}\left(t\right)$$

where *k*_2_ (g mg^–1^ min^–1^) represents the equilibrium rate constant for PSOM adsorption.

Assuming the applicability of the pseudo-second-order model (PSOM), a linear relationship is observed in the plot of *t/qt* versus *t*. From this linear trend, the rate constant *k*_*2*_ and the equilibrium adsorption capacity *q*_*e*_ can be determined from the slope and intercept, respectively, as illustrated in Figs. 13(b) and 14(b) for AY36 and MR dye adsorption onto GASC. Tables S3 and S5 summarize the PSOM-derived constants (*k*_*2*_), the theoretical and experimental *q*_*e*_ values, and the corresponding correlation coefficients (*R*^2^). The data analysis reveals that the *R*^2^ values for the PSOM closely approach unity, indicating a strong correlation between the calculated and experimental adsorption capacities across all tested initial dye concentrations. The mechanisms of organic dye adsorption mainly encompass physical adsorption, which includes electrostatic attraction, complexation, hydrogen bonding, π-π interactions, and pore-filling along with network entanglement. In acidic circumstances, the adsorption of anionic dyes by adsorbents is chiefly attributed to electrostatic interactions facilitated by protonated amino groups. In alkaline settings, elevated pH inhibits the protonation of -NH_3_, leading to a primarily chemical adsorption process^99^. These findings confirm that the PSOM provides an excellent fit for describing the adsorption kinetics of both AY36 and MR dyes onto GASC, making it the most appropriate kinetic model among those evaluated.

#### Elovich kinetic model (EM)

The removal of AY36 and MR dyes using GASC as an adsorbent was analyzed through the application of the Elovich Model (EM). Originally formulated for gas-phase adsorption, the EM has proven effective in modelling adsorption processes in liquid-phase systems such as wastewater treatment. This model provides insight into the energy dynamics associated with activation or deactivation processes, as well as mass and particle transport over solid surfaces. According to the theoretical basis established by Cheung et al. (2000)^100^ and Dotto and Pinto (2011)^101^, the rate of solute adsorption diminishes exponentially with an increase in the quantity of deposited solute. The Elovich Model is particularly useful for evaluating the heterogeneity of adsorption sites on the surface of the adsorbent. Its mathematical expression is presented in Eq. (14).

14 $$\:{q}_{\text{t}}=\frac{1}{\beta\:}\text{ln}\left(\alpha\:\beta\:\right)+\frac{1}{\beta\:}\text{l}\text{n}\left(t\right)$$

where *α* (mg g^–1^ min^–1^) and *β* (g mg^–1^) represent the initial sorption rate constant and the surface coverage, as well as the activation energy for chemisorption, respectively. The Elovich Model (EM) constants were determined by evaluating the slope and intercept of the linear plots of *q*_*t*_ versus ln *t*, as shown in Figs. 13(c) and 14(c). The corresponding calculated values are presented in Tables S4 and S6. For the adsorption of AY36 and MR dyes onto GASC, the correlation coefficients (*R*^2^) range from 0.769 to 0.989 and 0.957 to 0.999, respectively, indicating a reasonable fit between the model and the experimental data. A comparative analysis of the R^2^ values reveals that the EM provides a slightly better correlation than the pseudo-first-order model (PFOM), though it remains less accurate than the pseudo-second-order model (PSOM), as observed in Tables S3–S6. Overall, the results confirm that chemisorption plays a dominant role in the adsorption mechanism of AY36 and MR dyes onto the GASC adsorbent.

#### Intra-particle diffusion model (IPDM)

The adsorption process for removing pollutants using a solid-phase material typically involves four successive steps: (1) Pollutants are initially transported from the bulk solution to the external surface of the solid through bulk diffusion. (2) This is followed by film diffusion, whereby pollutants move across the boundary layer adjacent to the solid surface. (3) Subsequently, intraparticle or pore diffusion facilitates the migration of pollutants into the internal structure of the adsorbent, allowing interaction with internal surfaces and pores. (4) Finally, adsorption occurs at active sites on the adsorbent surface via chemical mechanisms such as ion exchange, chelation, or complexation^55^. The intraparticle diffusion model (IPDM) has been employed to examine the mass transfer mechanisms between the liquid phase and the adsorbent, as described by Kargi and Cikla^102^. In systems employing a batch setup with intensive agitation, isothermal plug flow diffusion (IPDM) can serve as the rate-limiting mechanism governing the adsorption process^103^. The analysis of IPDM in this context was further supported by applying Eq. (15), as originally introduced by Annadurai et al.^104^.

15 $$\:{q}_{t}={K}_{diff}{t}^{0.5}+C$$

where *K*_*diff*_ denotes the rate constant of IPDM (mg g^− 1^ min^1/2^) (Figs. 13(d) and 14(d)).

The model introduced by Weber and Morris^105^ suggests that intraparticle diffusion is the dominant mechanism in adsorption when the plot of *q*_*t*_ versus *t*^*0.5*^ passes through the origin, as illustrated in Figs. 14(d) and 14(d). Conversely, if the plot does not intersect the origin, particularly in cases with a large intercept (*C* value), film diffusion (FDM) is generally considered the primary rate-limiting step. This study examined the effectiveness of GASC in removing AY36 and MR dyes under various conditions, including different adsorbent dosages and initial dye concentrations. Figures 13(d) and 14(d) display the Weber–Morris^105^ intraparticle diffusion plots for the adsorption of AY36 and MR dyes, respectively. The corresponding diffusion rate constants (*K*_*dif*_) and intercepts (*C*) were calculated from the slopes and intercepts of the linear plots of *q*_*t*_ versus *t*^*0.5*^, as summarized in Tables S4 and S6. For both dyes, the plotted lines at various adsorbent concentrations do not intersect the origin, indicating the presence of a substantial intercept (*C* value). This deviation from the origin supports the conclusion that film diffusion (FDM) plays a significant role in controlling the adsorption rate of AY36 and MR dyes onto the GASC adsorbent. As illustrated in Figs. 13(e) and 14(e), the adsorption rate of AY36 and MR dyes onto GASC increases progressively over time. The intraparticle diffusion rate constant (*K*_*dif*_) was found to range from 0.42 to 6.47 mg g^–1^ min^–1/2^ for AY36 and from 0.18 to 5.90 mg g^–1^ min^–1/2^ for MR dye. A clear trend emerged, showing that *K*_dif_ increased with higher initial dye concentrations and lower GASC dosages. This behavior can be attributed to the gradual decline in available surface area and pore volume of the GASC adsorbent during the adsorption process.

#### Film diffusion model (FDM)

The film diffusion model (FDM) describes the transport of adsorbate molecules across the boundary layer surrounding the adsorbent particle^55^. This process is mathematically represented by Eq. (16).

16 $$\:\text{ln}\left(1-F\right)={K}_{FD}\left(t\right)$$

where *K*_FD_ denotes the external film mass transfer coefficient, and *F* signifies the ratio of *q*_t_ to *q*_e_. The film diffusion constant (*K*_*FD*_) was calculated by analyzing the slope and intercept of the linearized plot of (1 − F), as illustrated in Figs. 13(e) and 14(e)^106^. Among the evaluated kinetic models, the pseudo-second-order model (PSOM) demonstrated the best fit for describing the electrochemical adsorption of AY36 and MR dyes onto GASC. This conclusion is supported by the observation that the linear plots did not pass through the origin, indicating that film diffusion is not the rate-limiting step in the overall adsorption kinetics. Furthermore, the PSOM provided the highest correlation coefficient (*R*^2^ = 1), confirming its suitability for modelling the adsorption behavior. In the initial stage of the adsorption process, electrostatic interactions are presumed to occur between the negatively charged functional groups on the self-doped activated carbon and hydrogen ions in the solution. This mechanism is corroborated by the fitting results of the PSOM, LIM, and TIM isotherm models. The target compounds contain multiple nitrogen atoms with lone electron pairs, facilitating interaction with the adsorbent. The positively self-doped surface of GASC effectively adsorbed both AY36 and MR dyes, leading to the formation of a well-defined adsorption layer.

### Comparison with the result reported in the literature

A literature-based comparison was performed to evaluate the efficiency of various adsorbents in the removal of azo dyes, with particular emphasis on the performance of the GASC adsorbent. Table 4 provides a comparative assessment of adsorption capacities (mg/g) observed in this study alongside previously reported values from the literature. The findings clearly demonstrate that GASC effectively removes both AY36 and MR dyes, highlighting its potential as a competitive adsorbent.

**Table 4:** Comparison of the maximum biosorption capacities of GASC employed for removing AY36 and MR dyes.

Adsorbent	Pollutant	Q _m_ (mg/g)	Ref.
N-doping activated carbons from fish waste and sawdust	AY36 dye	232.56	^107^
Superabsorbent hydrogel of Poly(3-acrylamidopropyl)-trimethylammonium chloride-co-N, N-DMA	AY36 dye	199.96	^108^
Refused tea waste activated carbon	AY36 dye	71.97	^109^
AC from peanut shells	AY36 dye	66.70	^7^
Green nanoceria (GN) and GN–NH_2_ (AGN) from Prosopis julifora leaves extract	AY36 dye	26.95	^110^
Caraway seeds	MR dye	208.00	^111^
Fennel seeds	MR dye	135.00	^112^
Biogas Plant Waste	MR dye	113.00	^113^
Green tea leaves	MR dye	103.00	^114^
Marigold	MR dye	102.43	^115^
GASC	AY36 dye	216.45	This Study
GASC	MR dye	454.55	This Study

### RSM study

 The most thoroughly examined method is RSM, which is classified outside the realm of machine learning algorithms. Research comparing these data analysis approaches with AI models has demonstrated that the former yields more scientifically sound information, underpinned by statistical evaluations and uncertainty calculations^116^. Analysis of variance (ANOVA) was conducted on the selected model to evaluate its significance and to identify the factors affecting the removal efficiency. The ANOVA results are summarized in Tables S7 and S8^60–65^. The F-values indicate the relative importance of the studied factors and their interactions with the response; factors or interactions with F-values greater than 1 are considered influential. Based on the data presented in Tables S9 and S10, the initial concentrations of AY-36 and MR dyes exerted the most pronounced effect on removal efficiency. Terms with p-values below 0.05 were deemed statistically significant, and the overall model significance is confirmed by a p-value less than 0.0001. Moreover, for AY-36 dye, the model’s robustness is supported by the small difference between the adjusted R² (0.9866) and predicted R² (0.9063), which is less than 0.2. In another study similar to ours, Kumari and colleagues (2025) reported on the simulation of Methylene Blue (MB) dye adsorption-based separation using *Oryza sativa* straw biomass (OSSB). Using three different modeling approaches (artificial neural networks (ANN), adaptive neuro-fuzzy inference systems (ANFIS), and response surface methodology (RSM)), the regression coefficients obtained from process modeling showed that RSM (R^2^ = 0.9216), ANN (R^2^ = 0.8864), and ANFIS (R^2^ = 0.9589) accurately predicted MB adsorption removal^117^.

From the experimental results, the equations describing the removal percentage of AY-36 dye were derived (Eqs. 17 and 18):

17 $$\begin{aligned}{\rm Removal \: \% for\: coded\: factors = 78.958+12.933\:A -7.913B+5.888\:C + 3.398AB} \\{\rm + 0.330AC + 2.488BC - 3.033A^2 + 4.172B_2 - 4.345C^2}\end{aligned}$$

 

18 $$\begin{aligned}{\rm Removal \:\% for\: actual\: factors = - 76.911 + 0.355\: dose - 0.703\:dye\:conc + 0.221\: Time + 0.001\:dose \times dye\: conc +}\\ {\rm 0.000 dose\: \times\: Time + 0.001\: dye\: conc \times Time - 0.001\:dose^2 + 0.002\:dye\: conc^2 - 0.001\:Time^2}\end{aligned}$$

On the other hand, for MR dye, the model’s strength is further demonstrated by the difference between the adjusted *R*^2^ (0.9673) and predicted *R*^2^ (0.7709) being less than 0.2.

Based on the results obtained, the Eqs. (19, 20) for MR dye removal % was obtained:

19 $$\begin{aligned}{\rm Removal\: \% \:for\: coded\: factors = 91.895 + 15.397A - 1.433B + 5.854C -}\\ {\rm 1.187AB - 1.499AC + 0.286BC - 9.181A2-4.339B2 - 1.253C2}\end{aligned}$$

20 $$\begin{aligned} {\rm Removal\: \% for\: actual\: factors = - 10.100 + 1.135\: dose + 0.357\: dye\: conc\: + 0.201\: Time - 0.000\: dose\times dye\: conc\:} \\{\rm - 0.000\: dose \times Time + 0.000\: dye conc \times Time - 0.004\: dose^2 - 0.002\: dye\: conc^2 - 0.0003\:Time^2} \end{aligned}$$

**Fig. 15:**
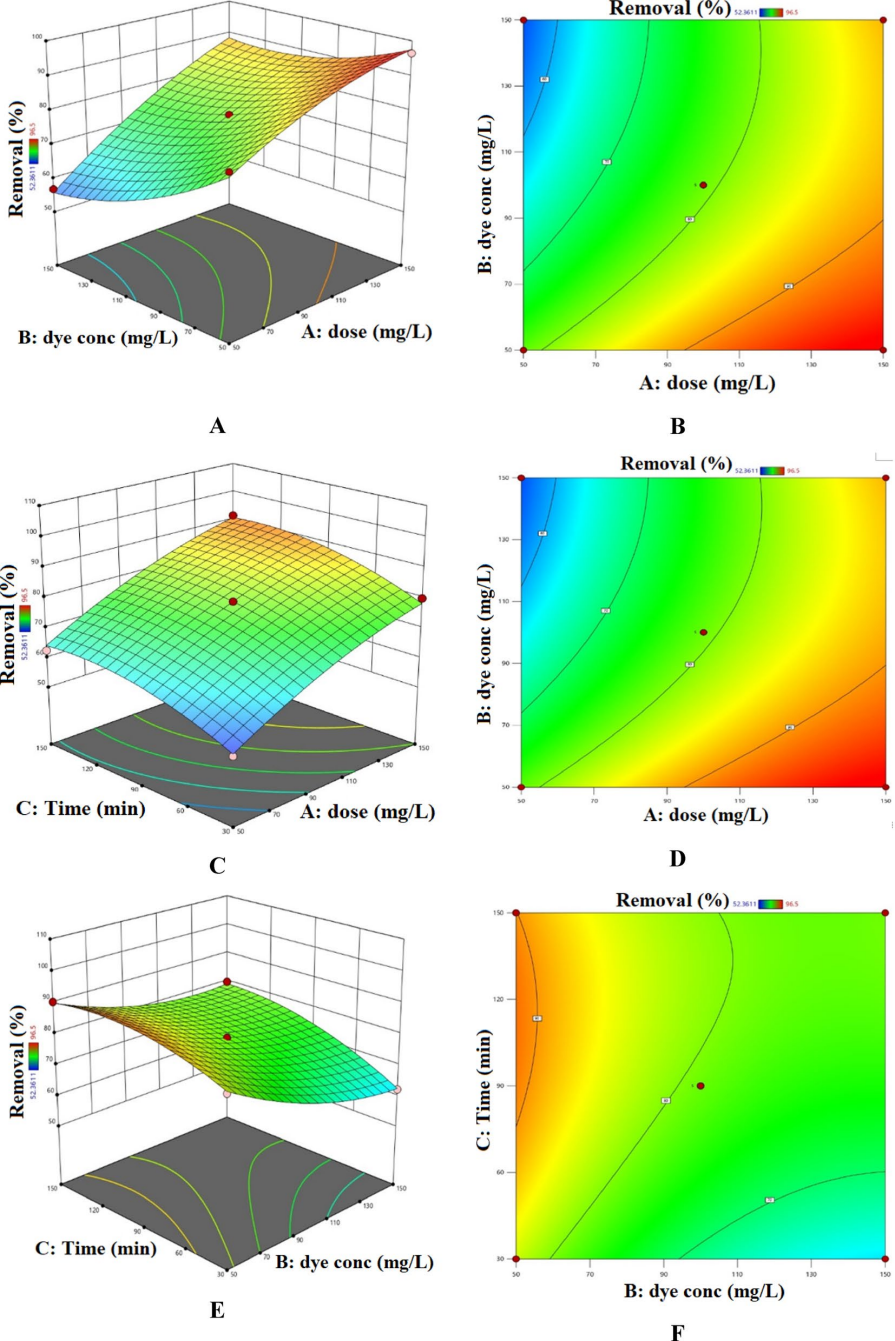

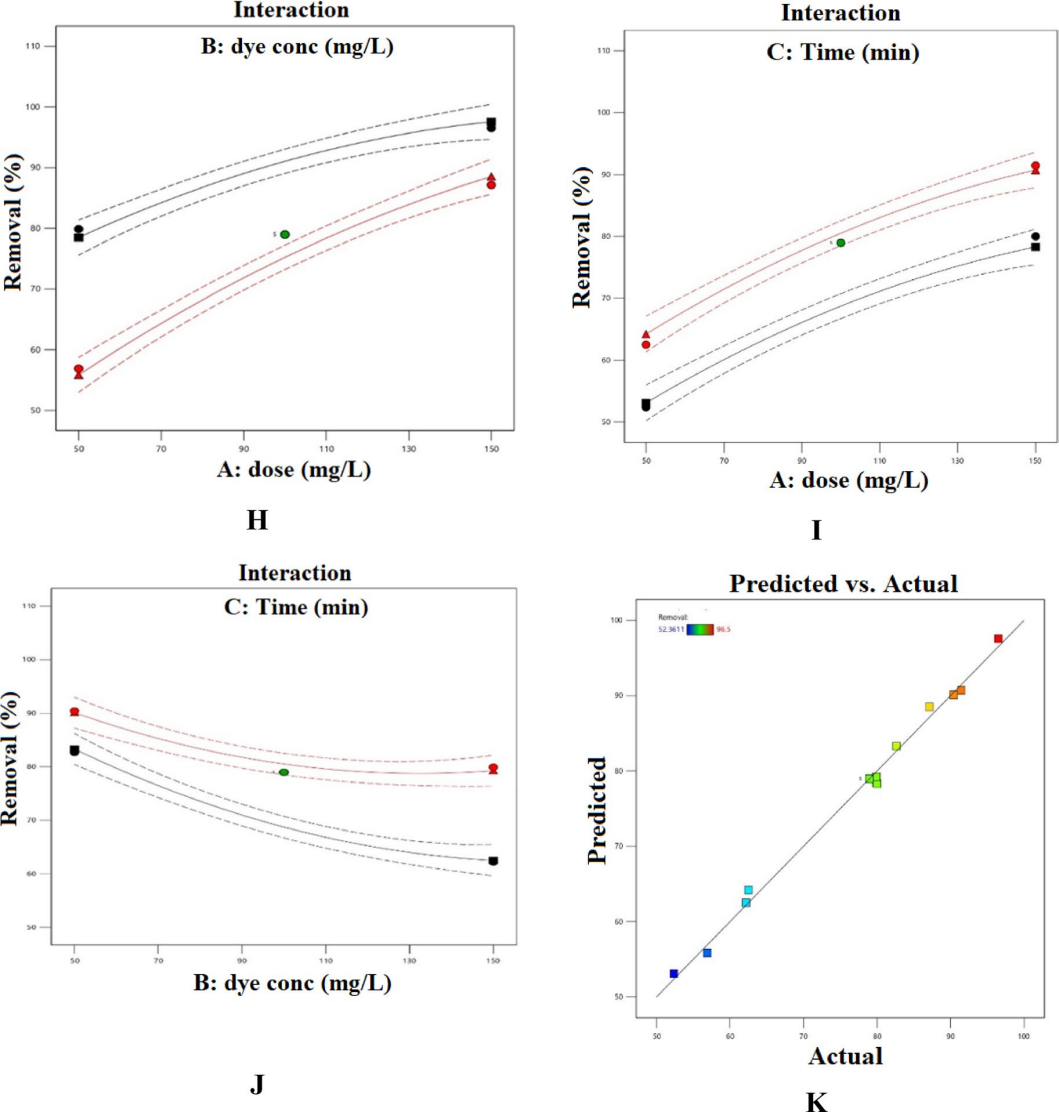
Combined influence of GASC dosage, AY-36 initial concentration, and contact time on adsorption: (**A, B**) dose–concentration, (**C, D**) dose–time, (**E, F**) concentration–time.

Figure 15 illustrates the combined influence of reaction time, adsorbent dosage, and initial AY-36 dye concentration on the removal efficiency of AY-36 dye. Optimal removal was achieved under conditions of low initial dye concentration, high GASC dosage, and extended contact time^64,65^.

**Fig. 16:**
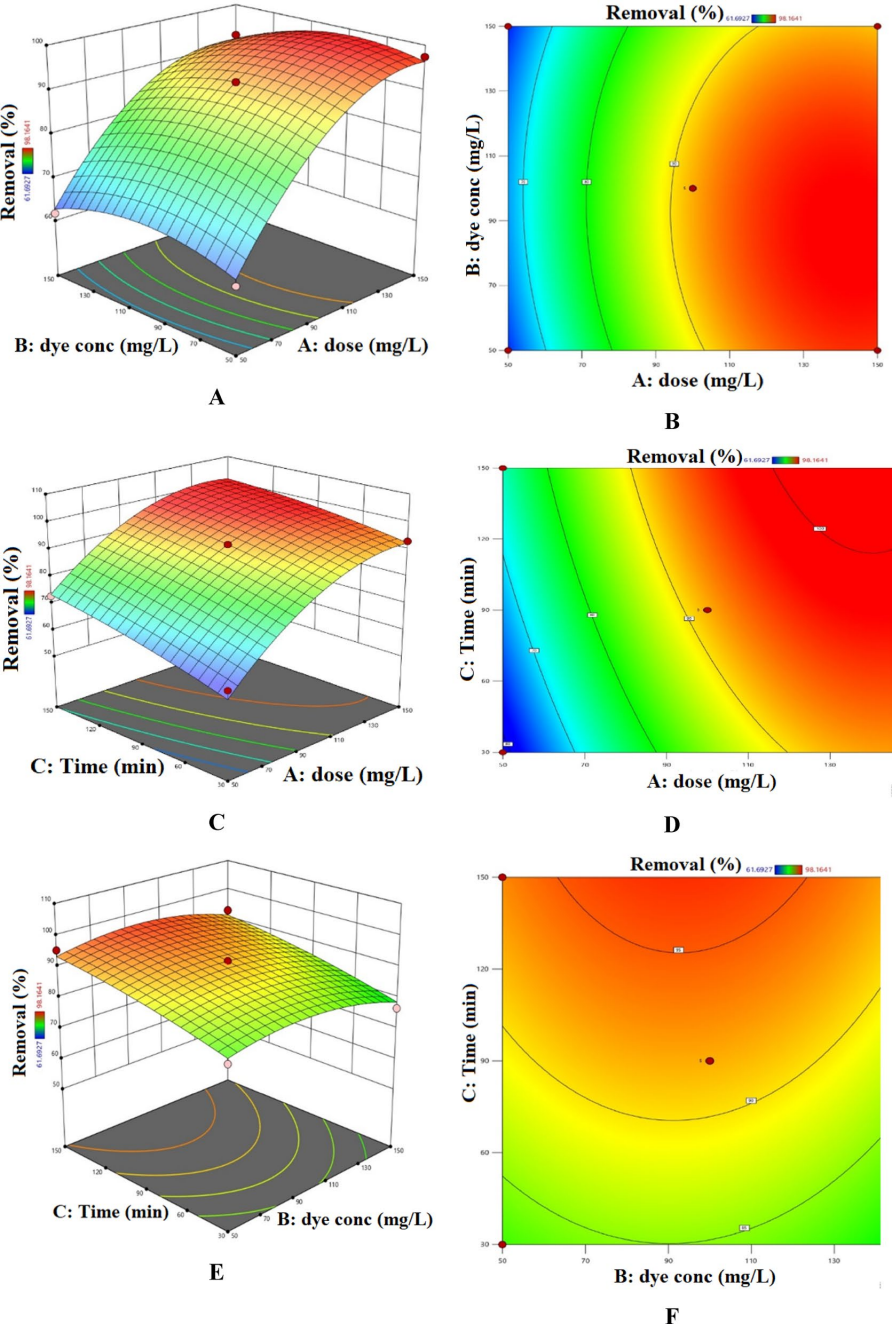

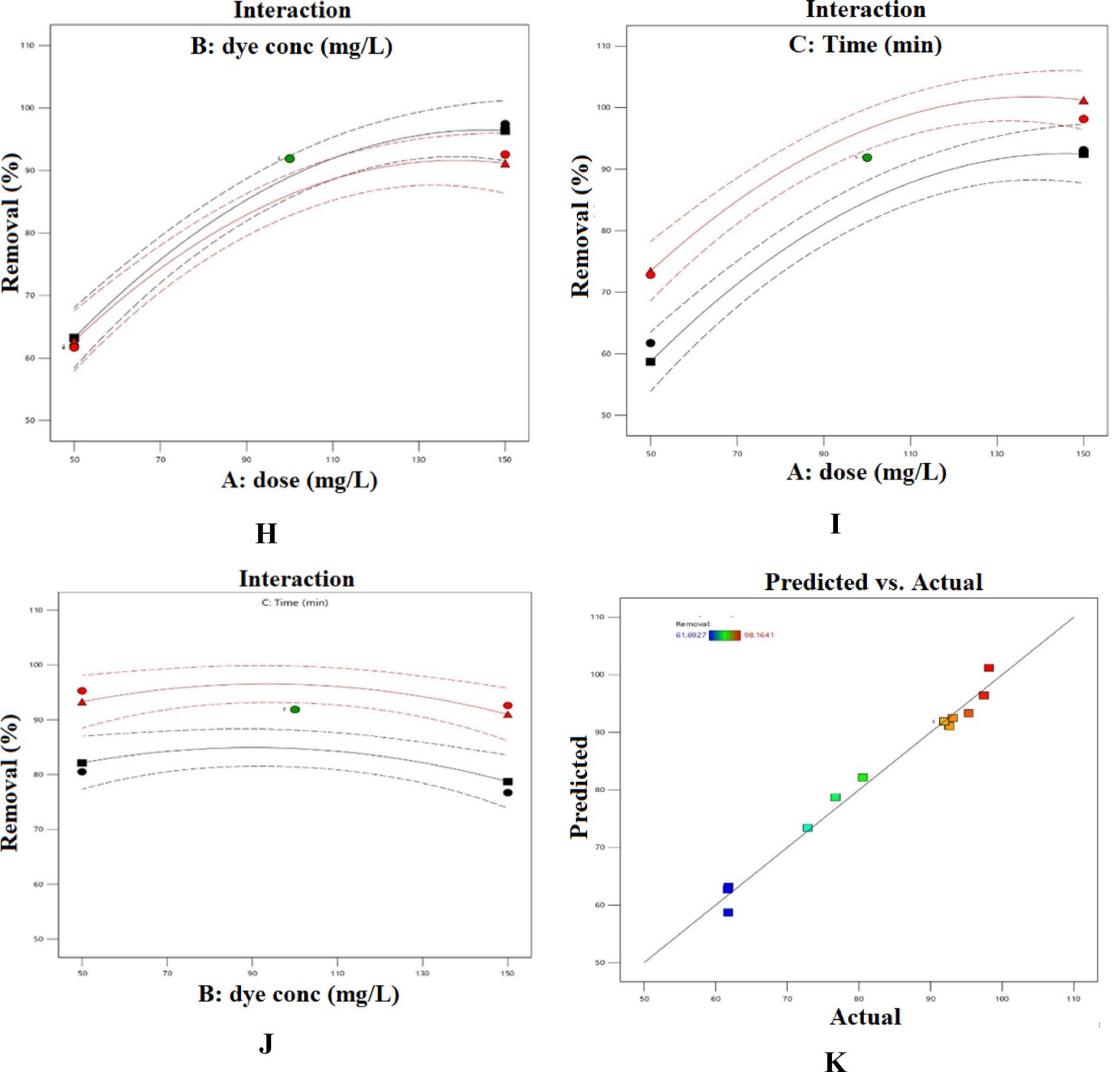
Combined influence of GASC dosage, MR initial concentration, and contact time on adsorption: (**A, B**) dose–concentration, (**C, D**) dose–time, (**E, F**) concentration–time.

Figure 16 illustrates the combined effects of reaction time, adsorbent dosage, and initial MR dye concentration on the removal efficiency of MR dye. Maximum removal was observed under conditions of low initial dye concentration, high GASC dosage, and prolonged contact time^64,65^.

**Fig. 17:**
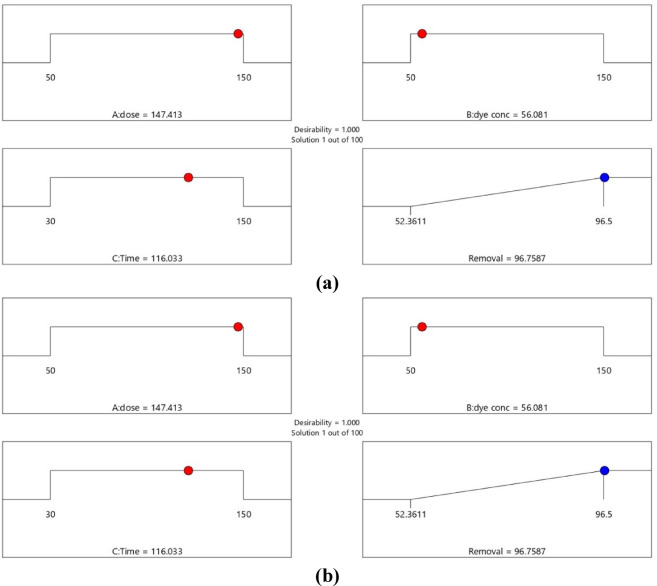
Optimization conditions through DOD settings (**a**) AY36 dye, (**b**) MR dye.

As illustrated in Fig. 17, the optimal operating conditions were determined numerically to achieve the highest removal efficiencies for both AY-36 and MR dyes.

### ANN study

**Fig. 18:**
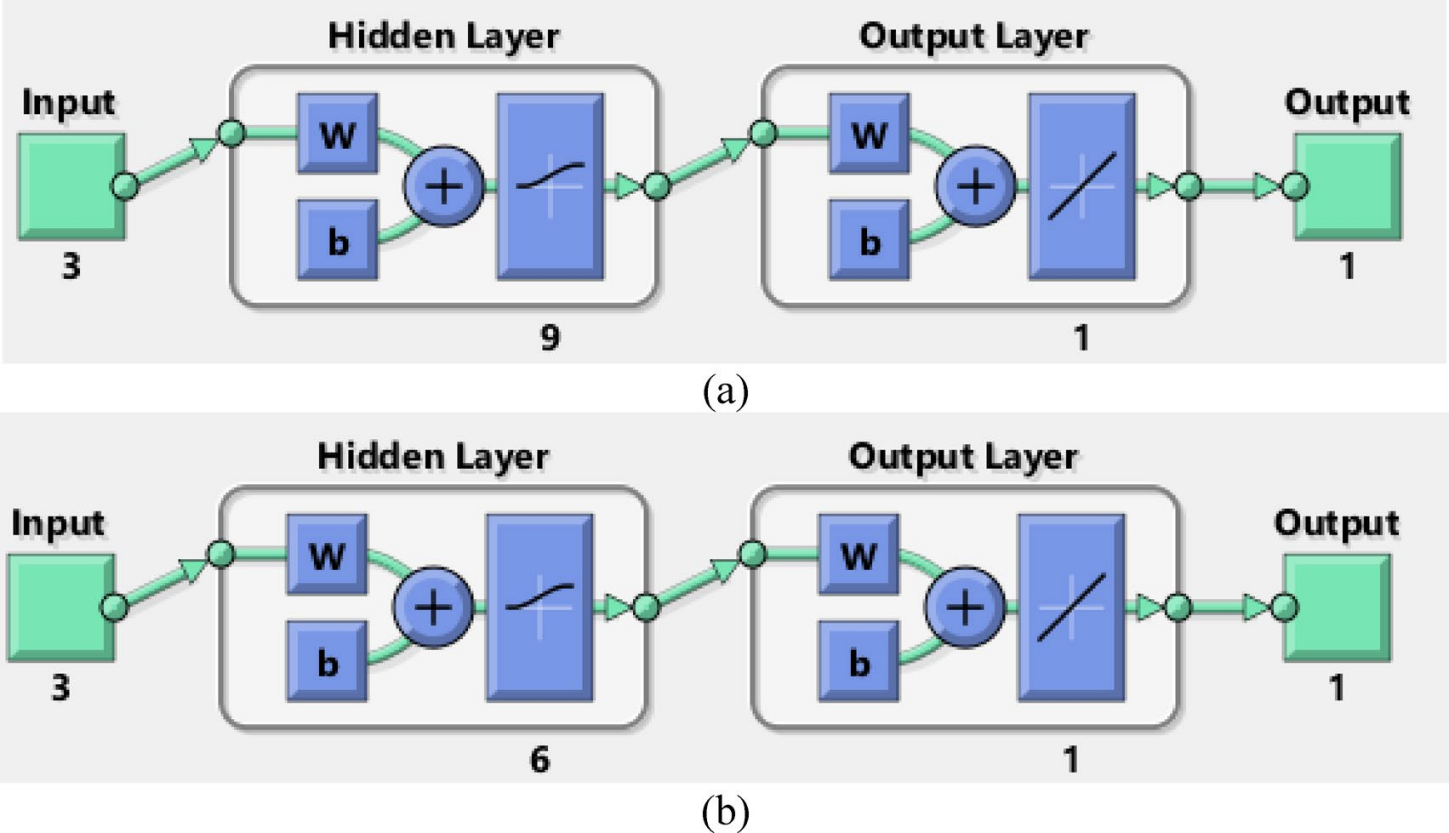
Schematic of ANN design applied to (**a**) AY36 and (**b**) MR dye adsorption.

**Fig. 19:**
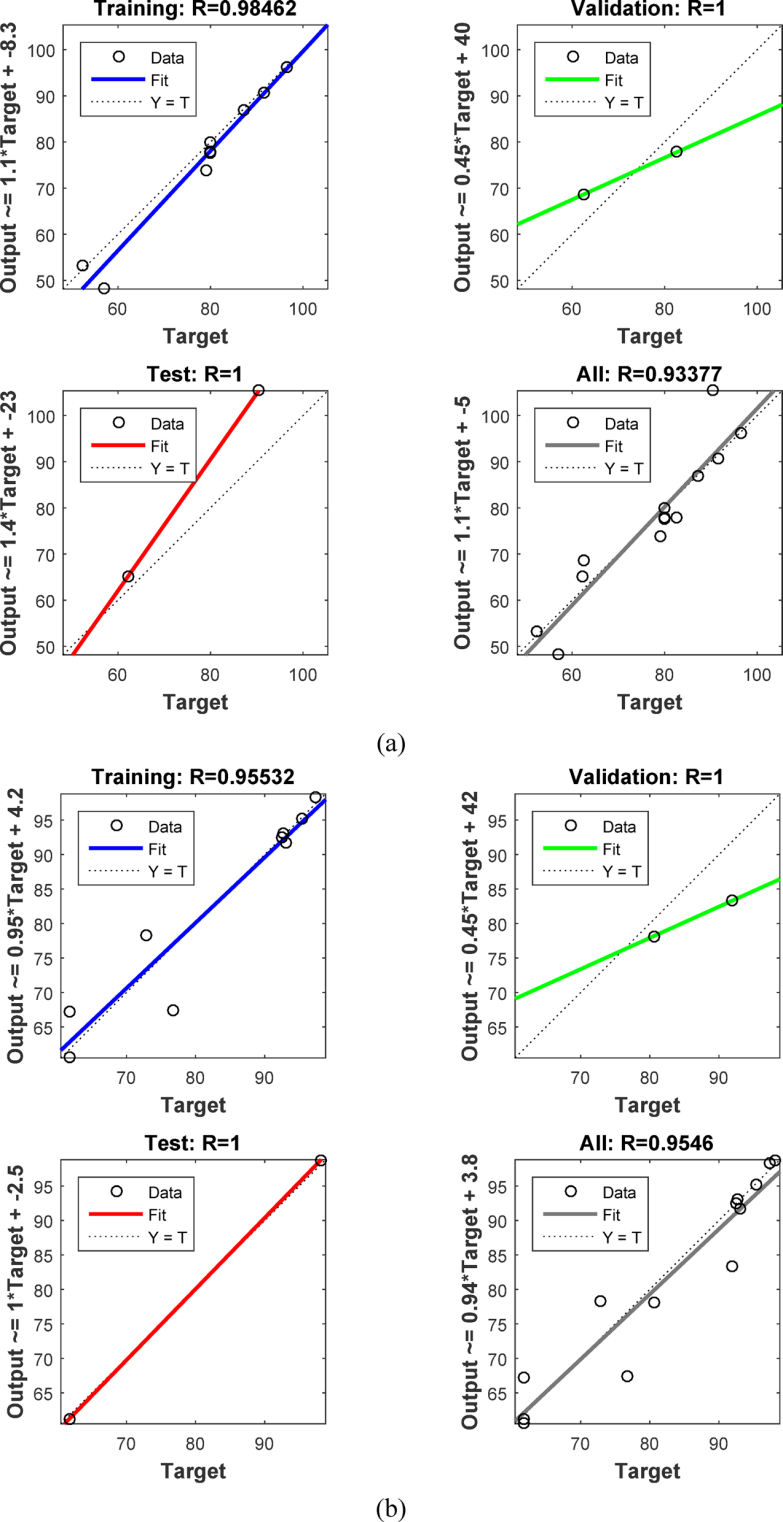
Training, validation, testing, and overall datasets for the LM algorithm for the removal of (**a**) AY 36 dye, and (**b**) MR dye.

**Fig. 20:**
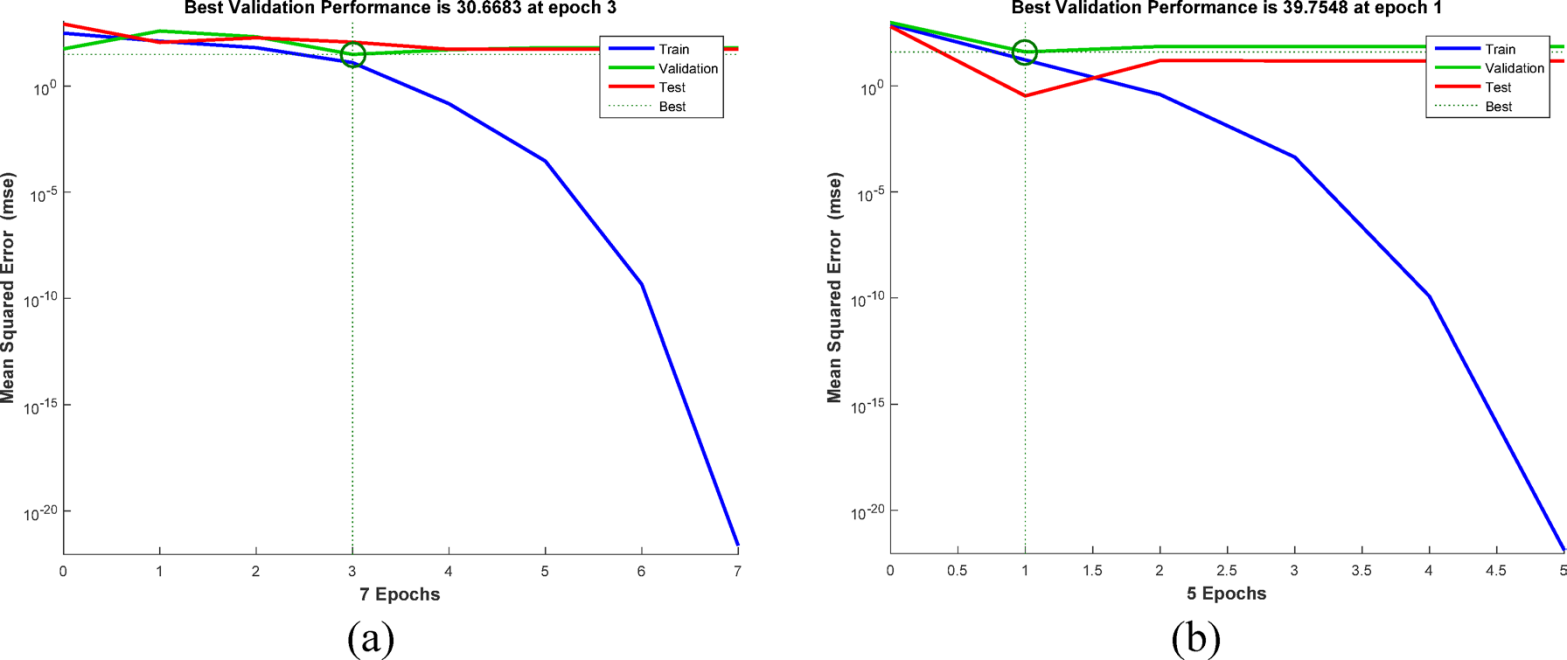
LM algorithm performance for the removal of (**a**) AY 36 dye, and (**b**) MR dye.

The conventional method of RSM exhibits certain limitations when characterizing behaviors beyond linear and quadratic correlations. In contrast, machine learning techniques such as artificial neural networks (ANN) deliver superior algorithmic performance and enhanced capacity to discern intricate correlations between input and output variables in diverse non-linear systems, hence augmenting predictive accuracy. Thus, data fitting and prediction can be accomplished via a well-trained artificial neural network model^118^. The ANN model was trained using the backpropagation algorithm. The network system for removing AY36 by the GASC optimal ANN model consists of 3 neurons in the input layer, 9 neurons in the hidden layer, and 1 neuron in the output layer, as shown in Fig. 18a. The regression plots in Fig. 19a shows that *R*^2^ training was 0.98462. *R*^2^ validation and testing were 1. *R*^2^ overall was 0.9338. The MSE value was 2.35e-22. The activation functions were log-sigmoid (log-sig) activation functions for the hidden layer and purelin for the output layer. The adsorbent dosage of GASC (g/L), time (min), and initial concentrations of AY36 are the three input variables of the optimal ANN. The optimal ANN output variable was the removal % of AY36 dye. Figure 20a displays the MSE error vs. the epoch number for the optimized ANN model that stopped after 3 epochs^119^. The network system for removing MR dye by the GASC optimal ANN model consists of 3 neurons in the input layer, 6 neurons in the hidden layer, and 1 neuron in the output layer, as shown in Fig. 18b. The regression plots in Fig. 19b show that *R*^2^ training was 0.95532. *R*^2^ validation and testing were 1. *R*^2^ overall was 0.9546. The MSE value was 1.31e-22. The activation functions were log-sigmoid (log-sig) activation functions for the hidden layer and purelin for the output layer. The adsorbent dosage of GASC (g/L), time (min), and initial concentrations of MR dye are the three input variables of the optimal ANN. The optimal ANN output variable was the removal % of MR dye. Figure 20b displays the MSE error vs. the epoch number for the optimized ANN model that stopped after 1 epoch^120^.

### Regeneration study

**Fig. 21:**
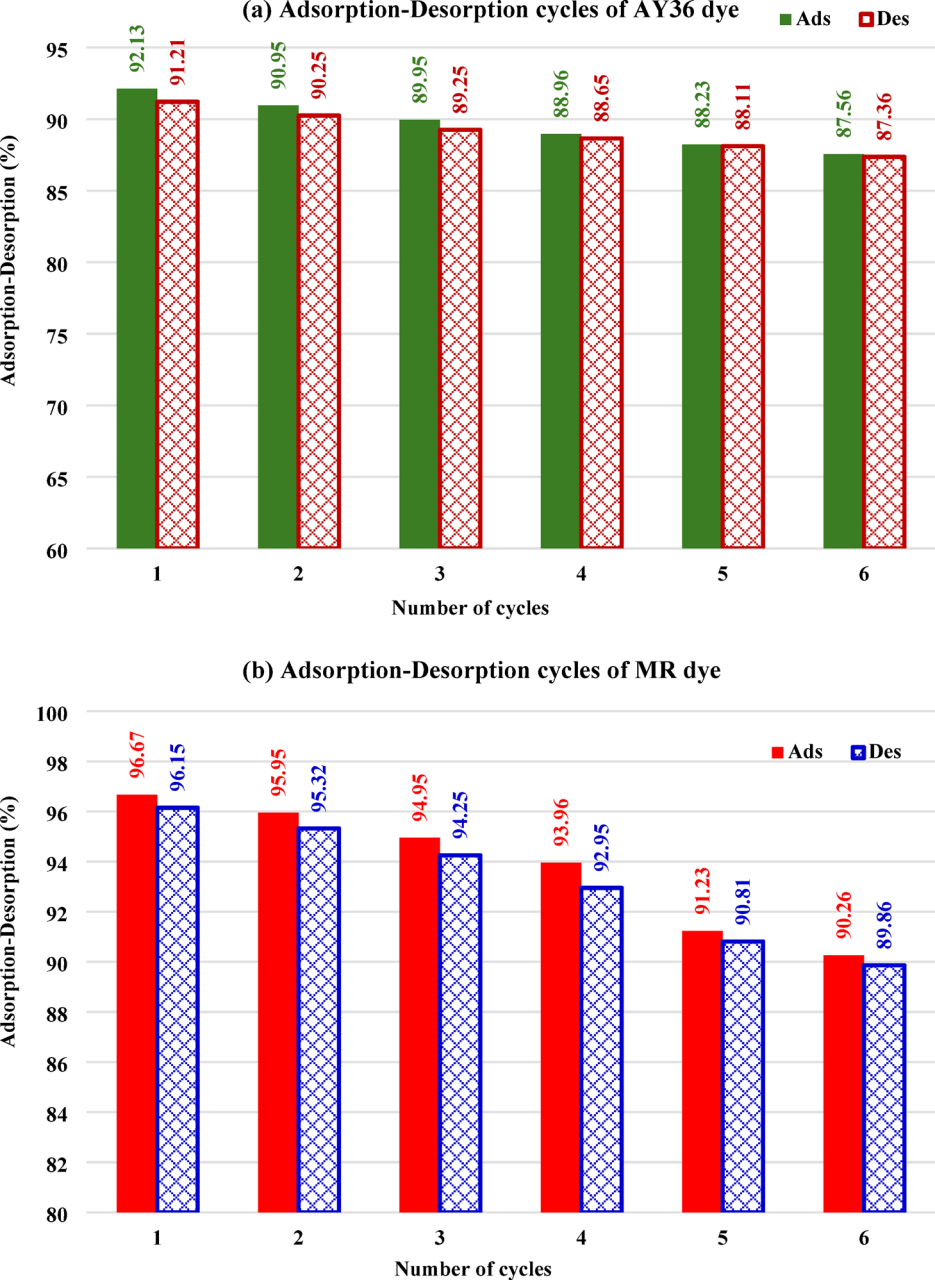
Regeneration study of (**a**) AY36 and (**b**) MR dye adsorption-desorption by GASC adsorbent using dye *C*_0_ (75 mg/g) and 1.5 g/L GASC dose at room temperature.

Using 0.1 M NaOH as an elution desorption medium and measuring its concentrations, desorption tests of the AY36 and MR dyes from the GASC adsorbent were conducted in order to examine the viability and adsorbent reusability for the absorption of AY36 and MR dyes. The GASC was then reactivated using 0.1 M HCl. The percentage of dye desorption in this study dropped as the regeneration cycles rose (Fig. 21). The regenerated GASC has been used to study six adsorption/desorption cycles. Adsorption and desorption changes were essentially constant across the cycles. After six cycles, though, it dropped by roughly 6.41% for MR dye and 4.57% for AY36 dye. It implies that GASC might be applied as a long-lasting method of removing AY36 dye and MR dye from water (Fig. 21)^121,122^. Thermodynamic investigation of AY36 and MR dye removal at different temperature would be inportant to have good information about the applicability of GASC adsorbent^123,124^. The techno-economic analysis will be very important to have the econmic valuable of the proposed adsorbent^125^.

## Conclusion

In this study, a novel sulfonated biochar carbon (GASC) derived from green algae (*Ulva lactuca*) was successfully synthesized using 80% sulfuric acid via the reflux method. This represents a sustainable and cost-effective approach for developing high-performance adsorbents from biomass, addressing environmental concerns related to both dye contamination and waste treatment. The sulfonation process was crucial in tailoring the surface chemistry of biochar and introduced functional groups that significantly enhanced the adsorption capabilities for anionic dyes such as Acid Yellow 36 (AY36) and Methyl Red (MR). This targeted modification represents a significant conceptual advance in the design of efficient adsorbents. The results of this study demonstrate the successful synthesis of activated carbon with high microporosity and specific surface area. Sulfonated biochar carbon (GASC) was developed using sulfuric acid as the activating agent through a reflux-based activation process. The adsorption behavior of AY36 and MR dyes onto GASC was investigated under optimized conditions. The study utilized a comprehensive suite of isotherm and kinetic models (LIM, FIM, TIM, DRIM, GIM, PFOM, PSOM, EM, IPDM, and FDM) to comprehensively understand the adsorption mechanisms. This integrated approach provides a deeper and more comprehensive perspective on the complex interactions between dyes and the adsorbent. Based on the Langmuir isotherm model, the maximum adsorption capacities (*Q*_m_) for AY36 and MR dyes were 216.45 and 454.55 mg g⁻¹, respectively. Among the kinetic models applied, the FIM model showed the best fit to the experimental data, indicating its suitability for describing the adsorption process of both dyes onto GASC. The adsorption kinetics of AY36 and MR dyes onto the GASC adsorbent were best described by the PSOM, indicating chemisorption as the rate-limiting step. Activated carbon synthesized from green algae represents a sustainable and effective approach for dye removal from aqueous media. The GASC exhibited notably high performance in eliminating both AY36 and MR dyes. The study uniquely combined Response Surface Methodology (RSM) and Artificial Neural Networks (ANN) to model and optimize the adsorption process. This synergistic use of advanced computational techniques provides a robust framework for estimating removal efficiencies and optimizing operational parameters, representing a significant methodological innovation for environmental engineering applications.The optimization of parameters was conducted using RSM, and the results concluded that a maximum AY-36 dye degradation percentage (96.76%) could be reached when employing 147.41 mg of the GASC adsorbent and 56.08 mg/L of AY-36 dye solution in 116 min. On the other hand, maximum MR dye degradation percentage (99.94%) could be reached when employing 132.34 g of the GASC adsorbent and 59.30 mg/L of MR dye solution in 140 min. The successful application of GASC for the high-efficiency removal of AY36 and MR dyes highlights its potential as an environmentally friendly and economically viable solution for industrial wastewater treatment. This contributes to broader goals such as sustainable resource management and pollution control. The detailed characterization and mechanistic insights gained from this study provide a strong foundation for the development of next-generation biochar-based adsorbents with improved selectivity and capacity for a variety of pollutants. The findings encourage further investigation of Ulva lactuca and similar biomasses as precursors for advanced functional materials.

## Supplementary Information

Below is the link to the electronic supplementary material.

Supplementary Material 1

## Data Availability

The datasets used in this study are available for review upon request from the corresponding author.
