# Supplementary material for: Modeling studies of the adsorption of Methyl Red and Acid Yellow 36 dyes by sulphonated Ulva lactuca carbon
Source: Sci Rep. 2025 Dec 10;15:43586. doi: 10.1038/s41598-025-29983-3 (PMC12698766; doi:10.1038/s41598-025-29983-3)
Supplement: Supplementary file 1 — Supplementary Material 1 [file 41598_2025_29983_MOESM1_ESM.docx]

**Supplementary materials**

**Modeling studies of the adsorption of Methyl Red and Acid Yellow 36 dyes by sulphonated *Ulva lactuca* Carbon**

Mohamed A. Hassaan^1^, Murat Yılmaz^2^, Amany El Sikaily^1^, Amany G. M. Shoaib^1^, Mohamed A. El-Nemr^3,4^, Ahmed El Nemr^1^*

^1^Environment Division, National Institute of Oceanography and Fisheries (NIOF), Kayet Bey, Elanfoushy, Alexandria, Egypt

^2^Osmaniye Korkut Ata University, Bahçe Vocational School, Department of Chemistry and Chemical Processing Technologies, Osmaniye, 80000, Türkiye

^3^Department of Chemical Engineering, Faculty of Engineering, Minia University, Minia 61519, Egypt

^4^The Higher Canal Institute of Engineering and Technology, Al Salam 1 - Abu Bakr Al Siddiq Street, Suez, Egypt

Email address: [mhss95@mail.com](mailto:mhss95@mail.com) (M.A. Hassaan); [muratyilmaz@osmaniye.edu.tr](mailto:muratyilmaz@osmaniye.edu.tr) (M. Yılmaz); [dramany_mas@yahoo.com](mailto:dramany_mas@yahoo.com) (A. El Sikaily); [amanygabershoaib@gmail.com](mailto:amanygabershoaib@gmail.com) (A.G.M. Shoaib); [mohamedelnemr1992@yahoo.com](mailto:mohamedelnemr1992@yahoo.com) (M.A. El-Nemr)

*Corresponding author: [ahmedmoustafaelnemr@yahoo.com](mailto:ahmedmoustafaelnemr@yahoo.com); [ahmed.m.elnemr@gmail.com](mailto:ahmed.m.elnemr@gmail.com)

**Table S1**. Adsorption isotherm analysis of AY36 dye adsorption by GASC at 25 °C.

| **Model** | **Parameters** | **GASC (g/L)** | | | | |
| --- | --- | --- | --- | --- | --- | --- |
|  |  | **0.50** | **0.75** | **1.00** | **1.25** | **1.50** |
| Linear LIM | *Q_m_* | 216.45 | 170.94 | 132.63 | 316.46 | 123.00 |
|  | *K_L_* x 10^3^ | 11.59 | 9.08 | 9.34 | 41.84 | 9.38 |
|  | *R*^2^ | 0.998 | 0.998 | 0.987 | 0.951 | 0.961 |
| Non-Linear LIM | *Q_m_* | 215.08 | 168.78 | 133.19 | 309.63 | 246.90 |
|  | *K_L_* x 10^3^ | 0.088 | 0.114 | 0.106 | 0.025 | 0.039 |
|  | *R*^2^ | 0.993 | 0.993 | 0.974 | 0.995 | 1.000 |
| FIM | *1/n* | 0.38 | 0.31 | 0.44 | 0.79 | 0.72 |
|  | *K_F_ (mg^1-1/n^L^1/n^g^–1^)* | 39.09 | 44.24 | 23.46 | 9.59 | 12.78 |
|  | *R*^2^ | 0.996 | 0.996 | 1.000 | 0.995 | 0.999 |
| TIM | *A_T_* | 1150 | 0.0272 | 2.14x10^32^ | 4.05x10^31^ | 3.29x10^61^ |
|  | *B_T_* | 0.02 | 0.03 | 0.02 | 0.02 | 0.01 |
|  | *b_T_* | 1.16x10^5^ | 9.18x10^4^ | 1.47x10^5^ | 1.45x10^5^ | 2.08x10^5^ |
|  | *R*^2^ | 0.989 | 0.997 | 0.989 | 0.999 | 0.908 |
| DRIM | *Q_m_ (mol kg^–1^)* | 140.70 | 111.43 | 105.90 | 116.42 | 98.31 |
|  | *K* × 10^6^ (mol kJ^–1^)^2^ | - | 2.80 | 9.00 | 9.80 | 5.30 |
|  | *E* (KJ mol^-1^) | 0.304 | 0.423 | 0.236 | 0.226 | 0.307 |
|  | *R*^2^ | 0.994 | 0.959 | 1.000 | 0.884 | 0.910 |
| GIM | *N_b_* | 0.62 | 0.56 | 0.56 | 1.00 | 0.92 |
|  | *K_G_* | 0.63 | 0.83 | 3.97 | 126.13 | 49.70 |
|  | *R^2^* | 0.980 | 0.985 | 1.000 | 0.996 | 1.000 |

**Table S2**. Adsorption isotherm analysis of MR dye adsorption by GASC at 25 °C

| **Model** | **Parameters** | **GASC (g/L)** | | | | |
| --- | --- | --- | --- | --- | --- | --- |
|  |  | **0.50** | **0.75** | **1.00** | **1.25** | **1.50** |
| Linear LIM | *Q_m_* | 454.55 | 196.08 | 181.82 | 129.87 | 113.64 |
|  | *K_L_* x 10^3^ | 72.55 | 8.55 | 5.60 | 1.25 | 0.94 |
|  | *R*^2^ | 0.887 | 0.987 | 0.940 | 0.968 | 0.988 |
| Non-Linear LIM | *Q_m_* | 513.49 | 194.37 | 175.54 | 143.27 | 100.42 |
|  | *K_L_* x 10^3^ | 0.011 | 0.097 | 0.198 | 0.331 | 1.115 |
|  | *R*^2^ | 0.950 | 0.587 | 0.884 | 0.162 | 0.532 |
| FIM | *1/n* | 0.72 | 0.48 | 0.58 | 0.91 | 0.42 |
|  | *K_F_ (mg^1-1/n^L^1/n^g^–1^)* | 10.97 | 30.43 | 32.03 | 21.11 | 51.14 |
|  | *R*^2^ | 0.991 | 0.998 | 0.903 | 0.995 | 1.000 |
| TIM | *A_T_* | 0.168 | 1.02 | 1.42 | 0.951 | 11.4 |
|  | *B_T_* | 86.973 | 45.441 | 44.414 | 61.848 | 23.91 |
|  | *b_T_* | 28.5 | 54.5 | 55.8 | 40.1 | 104 |
|  | *R*^2^ | 0.966 | 0.957 | 0.947 | 0.999 | 0.987 |
| DRIM | *Q_m_ (mol kg^–1^)* | 193.37 | 148.59 | 121.8 | 126.29 | 81.20 |
|  | *K* × 10^6^ (mol kJ^–1^)^2^ | 30.90 | 3.40 | 1.20 | 1.10 | 0.08 |
|  | *E* (KJ mol^-1^) | 0.127 | 0.383 | 0.645 | 0.674 | 2.5 |
|  | *R*^2^ | 0.975 | 0.983 | 1.000 | 0.995 | 0.992 |
| GIM | *N_b_* | 1.34 | 2.08 | 1.41 | 0.97 | 2.39 |
|  | *K_G_* | 8950 | 6.03x10^5^ | 3.9x10^4^ | 6.74x10^3^ | 5.9x10^6^ |
|  | *R^2^* | 0.944 | 0.908 | 0.714 | 0.833 | 0.976 |

**Table S3**. Estimated vs. Observed *q_e_* values and PFOM/PSOM adsorption rate constants for different AY36 dye and GASC concentrations

| **Parameter** | | |  | **PFOM** | | |  | **PSOM** | | | |
| --- | --- | --- | --- | --- | --- | --- | --- | --- | --- | --- | --- |
| **GASC**  **(g/L)** | **AY36 dye**  **(mg/L)** | ***q*_e_ (exp.)** |  | ***k*_1_ × 10^3^** | ***q*_e_ (calc.)** | ***R*^2^** |  | ***k*_2_ × 10^3^** | ***q*_e_**  **(calc.)** | ***h*** | ***R*^2^** |
| 0.50 | 50 | 84.72 |  | 16.35 | 24.10 | 0.980 |  | 2.15 | 85.47 | 15.72 | 0.999 |
|  | 75 | 121.94 |  | 24.87 | 53.25 | 0.917 |  | 1.08 | 125.00 | 16.95 | 0.997 |
|  | 100 | 135.00 |  | 9.44 | 37.23 | 0.623 |  | 1.10 | 133.33 | 19.53 | 0.994 |
|  | 125 | 166.11 |  | 11.75 | 51.65 | 0.964 |  | 0.83 | 166.67 | 23.04 | 0.997 |
|  | 150 | 182.36 |  | 22.57 | 81.77 | 0.980 |  | 0.62 | 188.68 | 22.22 | 0.998 |
| 0.75 | 50 | 60.28 |  | 19.58 | 15.39 | 0.916 |  | 3.85 | 60.98 | 14.33 | 0.999 |
|  | 75 | 87.87 |  | 18.42 | 30.39 | 0.988 |  | 1.72 | 89.29 | 13.70 | 0.998 |
|  | 100 | 109.68 |  | 24.87 | 41.31 | 0.865 |  | 1.47 | 112.36 | 18.52 | 0.999 |
|  | 125 | 128.33 |  | 22.11 | 55.13 | 0.854 |  | 1.00 | 131.58 | 17.39 | 0.997 |
|  | 150 | 142.50 |  | 17.27 | 55.72 | 0.991 |  | 0.85 | 144.93 | 17.86 | 0.998 |
| 1.00 | 50 | 45.42 |  | 26.25 | 8.34 | 0.956 |  | 8.82 | 45.87 | 18.55 | 1.000 |
|  | 75 | 64.86 |  | 14.05 | 16.30 | 0.984 |  | 2.81 | 65.79 | 12.18 | 0.998 |
|  | 100 | 84.79 |  | 27.64 | 39.40 | 0.862 |  | 1.71 | 86.96 | 12.95 | 0.998 |
|  | 125 | 98.19 |  | 18.65 | 40.15 | 0.939 |  | 1.19 | 101.01 | 12.17 | 0.996 |
|  | 150 | 122.78 |  | 18.65 | 45.09 | 0.944 |  | 1.08 | 126.58 | 17.30 | 0.998 |
| 1.25 | 50 | 35.78 |  | 15.43 | 4.65 | 0.965 |  | 11.42 | 36.10 | 14.88 | 1.000 |
|  | 75 | 53.06 |  | 20.27 | 8.37 | 0.953 |  | 8.02 | 53.48 | 22.94 | 1.000 |
|  | 100 | 72.28 |  | 31.78 | 21.22 | 0.980 |  | 3.74 | 74.07 | 20.53 | 1.000 |
|  | 125 | 87.66 |  | 17.96 | 23.14 | 0.936 |  | 2.44 | 88.50 | 19.08 | 0.999 |
|  | 150 | 103.44 |  | 37.77 | 53.05 | 0.931 |  | 1.50 | 107.53 | 17.39 | 0.999 |
| 1.50 | 50 | 28.38 |  | 56.65 | 6.33 | 0.951 |  | 24.35 | 28.49 | 19.76 | 1.000 |
|  | 75 | 46.06 |  | 20.50 | 4.45 | 0.896 |  | 16.29 | 46.08 | 34.60 | 1.000 |
|  | 100 | 60.97 |  | 28.56 | 15.03 | 0.945 |  | 4.91 | 62.11 | 18.94 | 1.000 |
|  | 125 | 73.75 |  | 28.56 | 15.47 | 0.933 |  | 4.74 | 74.63 | 26.39 | 1.000 |
|  | 150 | 90.09 |  | 25.33 | 33.25 | 0.991 |  | 1.87 | 92.59 | 16.05 | 0.999 |

**Table S4**. Adsorption rate constants of EM, IPDM, and FDM models at varying initial concentrations of AY36 dye and GASC.

| **GASC**  **(g/L)** | **AY36 dye**  **(mg/L)** |  | **EM** | | |  | **IPDM** | | |  | **FDM** | | |
| --- | --- | --- | --- | --- | --- | --- | --- | --- | --- | --- | --- | --- | --- |
|  |  |  | ***β*** | ***α*** | ***R*^2^** |  | ***K*_dif_** | ***C*** | ***R*^2^** |  | ***K*_FD_** | ***C*** | ***R*^2^** |
| 0.50 | 50 |  | 0.14 | 5.05x10^3^ | 0.974 |  | 2.19 | 57.29 | 0.953 |  | 0.016 | 1.26 | 0.980 |
|  | 75 |  | 0.09 | 4.85x10^3^ | 0.913 |  | 3.38 | 78.86 | 0.981 |  | 0.026 | 0.85 | 0.948 |
|  | 100 |  | 0.09 | 1.08x10^4^ | 0.769 |  | 3.36 | 85.73 | 0.933 |  | 0.009 | 1.17 | 0.974 |
|  | 125 |  | 0.07 | 6.88x10^3^ | 0.891 |  | 4.47 | 105.28 | 0.954 |  | 0.012 | 1.17 | 0.964 |
|  | 150 |  | 0.05 | 7.37x10^2^ | 0.920 |  | 6.47 | 103.12 | 0.937 |  | 0.023 | 0.80 | 0.980 |
| 0.75 | 50 |  | 0.23 | 2.70x10^4^ | 0.977 |  | 1.31 | 44.11 | 0.935 |  | 0.022 | 1.31 | 0.986 |
|  | 75 |  | 0.12 | 2.16x10^3^ | 0.989 |  | 2.48 | 56.63 | 0.978 |  | 0.019 | 1.06 | 0.988 |
|  | 100 |  | 0.09 | 1.06x10^3^ | 0.915 |  | 3.41 | 67.91 | 0.901 |  | 0.026 | 0.82 | 0.925 |
|  | 125 |  | 0.08 | 1.85x10^3^ | 0.952 |  | 3.85 | 79.22 | 0.968 |  | 0.014 | 1.11 | 0.948 |
|  | 150 |  | 0.07 | 1.01x10^3^ | 0.956 |  | 4.64 | 83.48 | 0.977 |  | 0.017 | 0.94 | 0.911 |
| 1.00 | 50 |  | 0.39 | 8.05x10^5^ | 0.968 |  | 0.81 | 40.76 | 0.815 |  | 0.026 | 1.70 | 0.956 |
|  | 75 |  | 0.21 | 2.05x10^4^ | 0.969 |  | 1.78 | 62.85 | 0.964 |  | 0.014 | 1.38 | 0.984 |
|  | 100 |  | 0.12 | 1.20x10^3^ | 0.968 |  | 3.23 | 59.43 | 0.985 |  | 0.018 | 1.09 | 0.962 |
|  | 125 |  | 0.10 | 1.04x10^3^ | 0.945 |  | 3.61 | 73.33 | 0.956 |  | 0.019 | 0.89 | 0.939 |
|  | 150 |  | 0.07 | 6.61x10^2^ | 0.959 |  | 1.85 | 111.60 | 0.961 |  | 0.019 | 1.00 | 0.944 |
| 1.25 | 50 |  | 0.73 | 1.51x10^9^ | 0.976 |  | 0.42 | 30.51 | 0.956 |  | 0.015 | 2.04 | 0.965 |
|  | 75 |  | 0.37 | 7.43x10^6^ | 0.970 |  | 0.79 | 43.37 | 0.900 |  | 0.021 | 1.84 | 0.953 |
|  | 100 |  | 0.17 | 8.55x10^3^ | 0.960 |  | 1.73 | 52.61 | 0.850 |  | 0.032 | 1.23 | 0.980 |
|  | 125 |  | 0.14 | 8.12x10^3^ | 0.974 |  | 2.15 | 61.27 | 0.911 |  | 0.018 | 1.33 | 0.936 |
|  | 150 |  | 0.08 | 5.45x10^2^ | 0.956 |  | 3.58 | 61.69 | 0.930 |  | 0.038 | 0.67 | 0.931 |
| 1.50 | 50 |  | 1.29 | 3.93x10^13^ | 0.935 |  | 0.67 | 17.15 | 0.914 |  | 0.013 | 2.56 | 0.825 |
|  | 75 |  | 0.61 | 1.87x10^10^ | 0.919 |  | 1.12 | 38.09 | 0.914 |  | 0.018 | 2.39 | 0.830 |
|  | 100 |  | 0.22 | 2.57x10^4^ | 0.952 |  | 1.33 | 53.01 | 0.969 |  | 0.029 | 1.40 | 0.945 |
|  | 125 |  | 0.19 | 5.19x10^4^ | 0.936 |  | 2.16 | 64.89 | 0.963 |  | 0.029 | 1.56 | 0.933 |
|  | 150 |  | 0.11 | 8.70x10^2^ | 0.986 |  | 2.48 | 74.64 | 0.981 |  | 0.025 | 0.99 | 0.991 |

**Table S5**. Comparison of predicted and experimental qₑ values for MR dye at varying initial concentrations and GASC dosages, along with PFOM and PSOM rate constants

| **Parameter** | | |  | **PFOM** | | |  | **PSOM** | | | |
| --- | --- | --- | --- | --- | --- | --- | --- | --- | --- | --- | --- |
| **GASC**  **(g/L)** | **MR dye**  **(mg/L)** | ***q*_e_ (exp.)** |  | ***k*_1_ × 10^3^** | ***q*_e_ (calc.)** | ***R*^2^** |  | ***k*_2_ × 10^3^** | ***q*_e_**  **(calc.)** | ***h*** | ***R*^2^** |
| 0.50 | 50 | 72.85 |  | 18.42 | 41.75 | 0.962 |  | 0.93 | 76.34 | 5.40 | 0.991 |
|  | 75 | 96.02 |  | 32.24 | 79.52 | 0.961 |  | 9.90 | 101.01 | 101.01 | 0.991 |
|  | 100 | 152.23 |  | 14.97 | 58.84 | 0.947 |  | 0.78 | 153.85 | 18.42 | 0.997 |
|  | 125 | 161.60 |  | 15.89 | 45.96 | 0.990 |  | 1.05 | 163.93 | 28.33 | 0.998 |
|  | 150 | 197.89 |  | 14.05 | 59.10 | 0.944 |  | 0.77 | 200.00 | 30.96 | 0.998 |
| 0.75 | 50 | 60.91 |  | 23.72 | 31.54 | 0.973 |  | 1.70 | 63.29 | 6.81 | 0.998 |
|  | 75 | 79.51 |  | 7.14 | 53.39 | 0.975 |  | 0.98 | 83.33 | 6.78 | 0.995 |
|  | 100 | 119.53 |  | 20.50 | 41.10 | 0.958 |  | 1.35 | 121.95 | 20.04 | 0.998 |
|  | 125 | 137.03 |  | 13.82 | 48.72 | 0.994 |  | 0.90 | 138.89 | 17.27 | 0.996 |
|  | 150 | 156.93 |  | 14.28 | 54.95 | 0.995 |  | 0.83 | 158.73 | 20.79 | 0.997 |
| 1.00 | 50 | 47.71 |  | 37.31 | 24.19 | 0.960 |  | 3.79 | 49.02 | 9.10 | 1.000 |
|  | 75 | 68.28 |  | 21.65 | 34.43 | 0.987 |  | 1.45 | 70.92 | 7.28 | 0.998 |
|  | 100 | 95.12 |  | 23.03 | 24.42 | 0.996 |  | 2.56 | 97.09 | 24.15 | 1.000 |
|  | 125 | 114.67 |  | 16.58 | 41.80 | 0.995 |  | 1.15 | 116.28 | 15.50 | 0.998 |
|  | 150 | 145.29 |  | 13.59 | 48.11 | 0.988 |  | 0.95 | 147.06 | 20.53 | 0.997 |
| 1.25 | 50 | 38.44 |  | 26.02 | 7.19 | 0.950 |  | 10.79 | 38.91 | 16.34 | 1.000 |
|  | 75 | 56.33 |  | 32.24 | 22.05 | 0.985 |  | 3.53 | 57.80 | 11.79 | 1.000 |
|  | 100 | 78.69 |  | 31.09 | 18.58 | 0.974 |  | 4.68 | 80.00 | 29.94 | 1.000 |
|  | 125 | 95.98 |  | 17.73 | 26.52 | 0.965 |  | 2.08 | 97.09 | 19.57 | 0.999 |
|  | 150 | 114.67 |  | 23.03 | 39.05 | 0.935 |  | 1.52 | 116.28 | 20,53 | 0.999 |
| 1.50 | 50 | 33.10 |  | 7.37 | 2.19 | 0.963 |  | 17.59 | 33.00 | 19.16 | 1.000 |
|  | 75 | 48.33 |  | 28.10 | 13.26 | 0.961 |  | 5.76 | 49.02 | 13.83 | 1.000 |
|  | 100 | 65.81 |  | 23.95 | 9.39 | 0.955 |  | 8.48 | 66.23 | 37.17 | 1.000 |
|  | 125 | 81.25 |  | 20.73 | 19.58 | 0.974 |  | 3.18 | 82.64 | 21.74 | 1.000 |
|  | 150 | 97.01 |  | 21.19 | 28.16 | 0.994 |  | 2.56 | 98.04 | 24.63 | 0.999 |

**Table S6**. Comparison of kinetic parameters (EM, IPDM, FDM) for MR dye adsorption on GASC under varying starting concentrations

| **GASC**  **(g/L)** | **MR dye**  **(mg/L)** |  | **EM** | | |  | **IPDM** | | |  | **FDM** | | |
| --- | --- | --- | --- | --- | --- | --- | --- | --- | --- | --- | --- | --- | --- |
|  |  |  | ***β*** | ***α*** | ***R*^2^** |  | ***K*_dif_** | ***C*** | ***R*^2^** |  | ***K*_FD_** | ***C*** | ***R*^2^** |
| 0.50 | 50 |  | 0.07 | 16.4 | 0.990 |  | 3.106 | 32.31 | 0.985 |  | 0.0189 | 0.565 | 0.979 |
|  | 75 |  | 0.07 | 50.1 | 0.960 |  | 4.060 | 42.67 | 0.986 |  | 0.0322 | 0.189 | 0.961 |
|  | 100 |  | 0.05 | 338 | 0.970 |  | 5.904 | 75.30 | 0.993 |  | 0.0157 | 0.820 | 0.990 |
|  | 125 |  | 0.06 | 2290 | 0.979 |  | 3.748 | 112.69 | 0.980 |  | 0.0160 | 1.257 | 0.990 |
|  | 150 |  | 0.06 | 13100 | 0.962 |  | 5.118 | 128.31 | 0.993 |  | 0.0135 | 1.155 | 0.997 |
| 0.75 | 50 |  | 0.13 | 109 | 0.993 |  | 2.356 | 32.82 | 0.981 |  | 0.0218 | 0.743 | 0.991 |
|  | 75 |  | 0.08 | 39.3 | 0.994 |  | 3.316 | 37.48 | 0.983 |  | 0.0172 | 0.679 | 0.992 |
|  | 100 |  | 0.09 | 2700 | 0.993 |  | 3.296 | 79.25 | 0.990 |  | 0.0167 | 1.194 | 0.996 |
|  | 125 |  | 0.08 | 2370 | 0.977 |  | 4.049 | 83.03 | 0.995 |  | 0.0139 | 1.034 | 0.994 |
|  | 150 |  | 0.07 | 2160 | 0.984 |  | 4.429 | 98.05 | 0.992 |  | 0.0143 | 1.049 | 0.995 |
| 1.00 | 50 |  | 0.20 | 445 | 0.983 |  | 1.660 | 29.02 | 0.951 |  | 0.0390 | 0.704 | 0.993 |
|  | 75 |  | 0.11 | 96 | 0.988 |  | 2.778 | 33.98 | 0.972 |  | 0.0220 | 0.690 | 0.990 |
|  | 100 |  | 0.14 | 24100 | 0.985 |  | 1.932 | 71.64 | 0.952 |  | 0.0231 | 1.360 | 0.996 |
|  | 125 |  | 0.09 | 1350 | 0.983 |  | 3.485 | 69.95 | 0.985 |  | 0.0166 | 1.009 | 0.995 |
|  | 150 |  | 0.07 | 2030 | 0.990 |  | 3.904 | 93.06 | 0.987 |  | 0.0131 | 1.159 | 0.995 |
| 1.25 | 50 |  | 2.55 | 1.7x10^28^ | 0.974 |  | 0.764 | 29.23 | 0.920 |  | 0.0264 | 1.545 | 0.976 |
|  | 75 |  | 0.17 | 591 | 0.970 |  | 1.964 | 35.36 | 0.889 |  | 0.0342 | 0.897 | 0.999 |
|  | 100 |  | 0.21 | 528000 | 0.983 |  | 1.564 | 61.72 | 0.903 |  | 0.0262 | 1.637 | 0.990 |
|  | 125 |  | 0.12 | 5680 | 0.991 |  | 2.441 | 65.38 | 0.953 |  | 0.0177 | 1.229 | 0.986 |
|  | 150 |  | 0.10 | 3470 | 0.999 |  | 2.568 | 81.80 | 0.974 |  | 0.0190 | 1.148 | 0.996 |
| 1.50 | 50 |  | 0.74 | 2.77x10^8^ | 0.960 |  | 0.183 | 30.32 | 0.920 |  | 0.0098 | 2.465 | 0.857 |
|  | 75 |  | 0.17 | 657 | 0.960 |  | 1.215 | 33.81 | 0.906 |  | 0.0240 | 1.425 | 0.940 |
|  | 100 |  | 0.30 | 9.88x10^6^ | 0.957 |  | 0.995 | 53.83 | 0.911 |  | 0.0271 | 1.895 | 0.954 |
|  | 125 |  | 0.15 | 12500 | 0.985 |  | 1.959 | 57.18 | 0.940 |  | 0.0207 | 1.390 | 0.983 |
|  | 150 |  | 0.12 | 6760 | 0.996 |  | 2.465 | 66.67 | 0.950 |  | 0.0212 | 1.237 | 0.994 |

**Table S7**. Experimental design for AY-36 dye removal using GASC adsorbent.

| **Run** | **Factor 1**  **A: Dose (g/L)** | **Factor 2**  **B: Conc. (mg/L)** | **Factor 3 C: Time (min)** | **Experimental**  **Removal %** | **Predicted**  **Removal %** |
| --- | --- | --- | --- | --- | --- |
| 1 | 1.00 | 100 | 90 | 78.96 | 78.96 |
| 2 | 0.50 | 50 | 90 | 79.86 | 78.48 |
| 3 | 1.50 | 100 | 30 | 80.00 | 78.30 |
| 4 | 1.00 | 100 | 90 | 78.96 | 78.96 |
| 5 | 1.00 | 50 | 150 | 90.42 | 90.10 |
| 6 | 1.00 | 150 | 30 | 62.18 | 62.49 |
| 7 | 1.00 | 100 | 90 | 78.96 | 78.96 |
| 8 | 1.00 | 100 | 90 | 78.96 | 78.96 |
| 9 | 0.50 | 100 | 30 | 52.36 | 53.09 |
| 10 | 1.50 | 50 | 90 | 96.50 | 97.55 |
| 11 | 0.50 | 150 | 90 | 56.90 | 55.85 |
| 12 | 1.50 | 150 | 90 | 87.13 | 88.52 |
| 13 | 1.00 | 100 | 90 | 78.96 | 78.96 |
| 14 | 0.50 | 100 | 150 | 62.50 | 64.20 |
| 15 | 1.00 | 50 | 30 | 82.64 | 83.30 |
| 16 | 1.00 | 150 | 150 | 79.91 | 79.25 |
| 17 | 1.50 | 100 | 150 | 91.46 | 90.73 |

**Table S8**. Experimental design for MR dye removal using GASC adsorbent.

| **Run** | **Factor 1**  **A: Dose (g/L)** | **Factor 2**  **B: Conc. (mg/L)** | **Factor 3 C: Time (min)** | **Experimental**  **Removal %** | **Predicted Removal %** |
| --- | --- | --- | --- | --- | --- |
| 1 | 1.00 | 100 | 90 | 91.89 | 91.89 |
| 2 | 0.50 | 50 | 90 | 61.80 | 63.22 |
| 3 | 1.50 | 100 | 30 | 93.09 | 92.50 |
| 4 | 1.00 | 100 | 90 | 91.89 | 91.89 |
| 5 | 1.00 | 50 | 150 | 95.31 | 93.30 |
| 6 | 1.00 | 150 | 30 | 76.72 | 78.73 |
| 7 | 1.00 | 100 | 90 | 91.89 | 91.89 |
| 8 | 1.00 | 100 | 90 | 91.89 | 91.89 |
| 9 | 0.50 | 100 | 30 | 61.76 | 58.71 |
| 10 | 1.50 | 50 | 90 | 97.43 | 96.39 |
| 11 | 0.50 | 150 | 90 | 61.69 | 62.73 |
| 12 | 1.50 | 150 | 90 | 92.58 | 91.15 |
| 13 | 1.00 | 100 | 90 | 91.89 | 91.89 |
| 14 | 0.50 | 100 | 150 | 72.83 | 73.42 |
| 15 | 1.00 | 50 | 30 | 80.55 | 82.17 |
| 16 | 1.00 | 150 | 150 | 92.63 | 91.01 |
| 17 | 1.50 | 100 | 150 | 98.16 | 101.21 |

**Table S9**. F and p-values were obtained from the D-Optimal design for key factors affecting AY36 dye removal

| **Source** | **Sum of Squares** | **df** | **Mean Square** | **F-value** | **p-value** |  |
| --- | --- | --- | --- | --- | --- | --- |
| **Model** | 2372.28 | 9 | 263.59 | 132.07 | < 0.0001 | **significant** |
| A-Adsorbent dosage | 1338.19 | 1 | 1338.19 | 670.48 | < 0.0001 |  |
| B- AY-36 Conc. | 500.95 | 1 | 500.95 | 250.99 | < 0.0001 |  |
| C-Time | 277.38 | 1 | 277.38 | 138.98 | < 0.0001 |  |
| AB | 46.19 | 1 | 46.19 | 23.14 | 0.0019 |  |
| AC | 0.4352 | 1 | 0.4352 | 0.2181 | 0.6547 |  |
| BC | 24.77 | 1 | 24.77 | 12.41 | 0.0097 |  |
| A² | 38.73 | 1 | 38.73 | 19.41 | 0.0031 |  |
| B² | 73.28 | 1 | 73.28 | 36.72 | 0.0005 |  |
| C² | 79.51 | 1 | 79.51 | 39.84 | 0.0004 |  |
| **Std. Dev.** | 1.41 | **R²** | 0.9941 |  |  |  |
| **Mean** | 77.45 | **Adjusted R²** | 0.9866 |  |  |  |
| **C.V. %** | 1.82 | **Predicted R²** | 0.9063 |  |  |  |
| **Adeq Precision** | 41.0305 |  |  |  |  |  |

**Table S10**. F and p-values obtained from D-Optimal design for key factors affecting MR dye removal.

| **Source** | **Sum of Squares** | **df** | **Mean Square** | **F-value** | **p-value** |  |
| --- | --- | --- | --- | --- | --- | --- |
| **Model** | 2672.25 | 9 | 296.92 | 53.54 | < 0.0001 | **significant** |
| A-Adsorbent dosage | 1896.62 | 1 | 1896.62 | 341.99 | < 0.0001 |  |
| B-Dye Conc. | 16.43 | 1 | 16.43 | 2.96 | 0.1288 |  |
| C-Time | 274.12 | 1 | 274.12 | 49.43 | 0.0002 |  |
| AB | 5.63 | 1 | 5.63 | 1.02 | 0.3470 |  |
| AC | 8.99 | 1 | 8.99 | 1.62 | 0.2436 |  |
| BC | 0.3282 | 1 | 0.3282 | 0.0592 | 0.8148 |  |
| A² | 354.92 | 1 | 354.92 | 64.00 | < 0.0001 |  |
| B² | 79.27 | 1 | 79.27 | 14.29 | 0.0069 |  |
| C² | 6.61 | 1 | 6.61 | 1.19 | 0.3109 |  |
| **Std. Dev.** | 2.35 | **R²** | 0.9857 |  |  |  |
| **Mean** | 84.94 | **Adjusted R²** | 0.9673 |  |  |  |
| **C.V. %** | 2.77 | **Predicted R²** | 0.7709 |  |  |  |
| **Adeq Precision** | 23.5316 |  |  |  |  |  |
